# Mechanism of MicroRNA-Target Interaction: Molecular Dynamics Simulations and Thermodynamics Analysis

**DOI:** 10.1371/journal.pcbi.1000866

**Published:** 2010-07-29

**Authors:** Yonghua Wang, Yan Li, Zhi Ma, Wei Yang, Chunzhi Ai

**Affiliations:** 1 Center of Bioinformatics, Northwest A&F University, Yangling, Shaanxi, China; 2 School of Chemical Engineering, Dalian University of Technology, Dalian, Liaoning, China; 3 Lab of Pharmaceutical Resource Discovery, Dalian Institute of Chemical Physics, Chinese Academy of Sciences, Dalian, Liaoning, China; University of Maryland, United States of America

## Abstract

MicroRNAs (miRNAs) are endogenously produced ∼21-nt riboregulators that associate with Argonaute (Ago) proteins to direct mRNA cleavage or repress the translation of complementary RNAs. Capturing the molecular mechanisms of miRNA interacting with its target will not only reinforce the understanding of underlying RNA interference but also fuel the design of more effective small-interfering RNA strands. To address this, in the present work the RNA-bound (Ago-miRNA, Ago-miRNA-target) and RNA-free Ago forms were analyzed by performing both molecular dynamics simulations and thermodynamic analysis. Based on the principal component analysis results of the simulation trajectories as well as the correlation analysis in fluctuations of residues, we discover that: 1) three important (PAZ, Mid and PIWI) domains exist in Argonaute which define the global dynamics of the protein; 2) the interdomain correlated movements are so crucial for the interaction of Ago-RNAs that they not only facilitate the relaxation of the interactions between residues surrounding the RNA binding channel but also induce certain conformational changes; and 3) it is just these conformational changes that expand the cavity of the active site and open putative pathways for both the substrate uptake and product release. In addition, by thermodynamic analysis we also discover that for both the guide RNA 5′-end recognition and the facilitated site-specific cleavage of the target, the presence of two metal ions (of Mg^2+^) plays a predominant role, and this conclusion is consistent with the observed enzyme catalytic cleavage activity in the ternary complex (Ago-miRNA-mRNA). Our results find that it is the set of arginine amino acids concentrated in the nucleotide-binding channel in Ago, instead of the conventionally-deemed seed base-paring, that makes greater contributions in stabilizing the binding of the nucleic acids to Ago.

## Author Summary

One of the biggest surprises at the beginning of the ‘post-genome era’ was the discovery of numerous genes encoding microRNAs. The number of microRNA genes is estimated to be nearly 1% of that of protein-coding genes, which were found in genomes of such diverse organisms as *Caenorhabditis elegans*, *Drosophila melanogaster*, *Arabidopsis thaliana*, and *Homo sapiens*. Their products, tiny RNAs (miRNAs and siRNAs), are thought to bind to Argonaute (Ago) proteins and form effector complexes to direct mRNA cleavage or repress translation of complementary RNAs, during development, organogenesis, and very likely during many other processes. The cellular interactions between the miRNAs and their target RNAs associating with Ago are only beginning to be revealed, and details of this interaction mechanism at molecular level are still poorly understood. In this article we propose the possible mechanisms of miRNA-target interaction with special emphasis on their structural dynamic and thermodynamic aspects. The results of our model suggest the chemical and physical factors and effects that may be responsible for the miRNA-Ago assembly and miRNA-target recognition.

## Introduction

As single-stranded RNA molecules of ∼21–23 nucleotide (nt) RNAs, microRNAs (miRNAs) post-transcriptionally regulate the eukaryotic gene expression by reducing the protein yield from specific target mRNAs, which function is crucial for control of a multitude of critical processes in both plant and animal cells [1], [2]. Comprising approximately 1% of genes in animals, miRNAs are often highly conserved across a wide range of species [3]. In animals, the functions of miRNA are always associated with regulation of many important processes, including the signaling pathways, apoptosis, metabolism, cardiogenesis and brain development (reviewed in Ref. 3). In addition, various types of cancers are also discovered as being probably the results of a misregulation of miRNA's expression (reviewed in Ref. 3). These facts all highlight the importance of miRNAs for both normal cell development and corresponding disease treatment as potential drug target.

In both animals and plants, miRNAs recognize their targets through a “seed” sequence. The core of the seed sequence resides between nucleotides 2–7 measured from the 5′-end of the guide strand, which sometimes, to a less extent, still includes the nucleotide 8 [4]. Wealth of data reported for the sequences of animal miRNAs and their targets have illustrated the key role of the seed region of complementarity [1], [5]. However, some miRNA–target site interactions found in the *C. elegans* [6], human cell [7] as well as mice cell [8] often violate the seed rule. It has been revealed that single-nucleotide changes beyond seed and central pairing were important for the miRNA-target recognition in planta [9]. Moreover, the seed match contains both a bulge and a GU wobble, and several other examples of plant miRNA–mRNA interactions with poor seed matches exist [10]. Clearly, only focusing on the seed, even with additional helper parameters, such as the compensatory 3′ pairing and AU-rich sequence occurrence around target sites, is not sufficient, and a better appreciation of the physical chemistry that underlies the target selection is still needed [11].

MiRNA functions by being captured into an argonaute-containing effector complex which is known as RISC (RNA induced silencing complex). As signature component of RISC [12]–[14], Ago proteins composed of PAZ- and PIWI-containing modules possess a central role in mediating distinct assembly and cleavage steps of the RNA interference catalytic cycle [15]. Structural and biochemical analyses have shown that the ∼130-amino-acid PAZ domain contains an oligonucleotide-binding fold that allows the protein to bind the single-stranded 2-nt 3′ terminal overhangs characteristic of small RNAs processed by Dicer [16]. Actually, the PAZ domain is composed of two subdomains with a cleft in between, where one subdomain consists of a five stranded open β-barrel with two helices on one end of the barrel, and the other one is made up of a β-hairpin followed by an α-helix. These two subdomains have been demonstrated be capable of RNA recognition [17], yet their dynamic properties involving in the single-stranded nucleic acid binding at atomic level still remain unclear.

As to another module of Ago proteins, i.e., the PIWI domain, it is located at the C-terminus of argonaute across the primary groove from the PAZ domain. Its core fold belongs to the RNase H family of enzymes, containing two highly conserved aspartates on adjacent three β-strands surrounded by α helices [18]. The reported *archaeal* crystal structure, together with the mutational analysis results, have revealed the necessity of two aspartate residues (D557 and D669) and one histidine residue (H807) for the catalysis of human Ago2 protein [19]. As seen from the crystal structure of *Thermus thermophilus* argonaute bound to a guide DNA and a target RNA at 3.0 Å resolution [20], a large nucleic-acid-binding channel is observed existing between the PAZ- and PIWI-containing lobes to accommodate the bound ligands. The seed segment adopts an A-helical-like Watson–Crick paired duplex, with both ends of the guide strand anchored in the complex [21]. It is considered that the PAZ and Mid domains of Ago proteins bind to the small RNA 3′ and 5′ ends, respectively, thus in this way they accurately fixed the position of the PIWI-mediated endonucleolytic cleavage of the target mRNA [19].

Recent reports have demonstrated that the RNA-induced silencing complex is an Mg^2+^-dependent endonuclease in both humans and flies [22], and the cleavage catalysis is mediated by the PIWI domain of Ago and occurs specifically 10 nt from the 5′-end of the miRNA, leaving the miRNA intact for another round of cleavage [23], [24]. Very recently, two Mg^2+^ cations were identified from the crystal structure of Ago complex (3F73.pdb [20]), with one cation bound to the catalytic triad (D478, D546 and D660) of the RNase H fold of the PIWI domain, the catalytic site for mRNA cleavage, yet the other coordinated by Val678 embeded in the Mid domain which might be involved in the anchoring of 5′ end phosphate of miRNA. It is speculated that Mg^2+^ ion might bind to the RNA substrate through a nonbridging oxygen of the scissile phosphate during catalysis, however, the detailed mechanism concerning the Mg^2+^ function in the process of RISC-mediated target RNA recognition still remains unknown [23]. In addition, although recent works have provided some insights into the miRNA-target interactions [20], [21], several fundamental questions are still open, including particularly: 1) how does Ago recognize the miRNA or mRNA dynamically? 2) What causes the conformational changes of miRNA or mRNA within the binding site? 3) How much does each Ago residue or RNA nucleotide contribute to the binding affinity? 4) What are the physical interactions dominating between the target mRNA and the receptor residues? 5) What conformational events and changes in metal binding may occur in proceeding to the transition state from a precatalytic active conformation?

To find possible answers to these questions, in the present work, molecular dynamics (MD) simulations were performed on the free Ago (single), miRNA-Ago (binary) and miRNA-mRNA-Ago (ternary) complexes. The purpose of these large scale simulations is to complement the experiments for better understanding of the miRNA molecular recognition mechanism by providing atomic details that are often inaccessible in experiments due to resolution limits. In addition, to deeply investigate the interactions between miRNA and its target, the principal component analysis (PCA) and thermodynamic analyses using molecular mechanics Poisson-Boltzmann surface area (MM-PBSA) were also applied. Our modeling and MD results for exploring the dynamic and thermodynamic mechanism of the miRNA-target interaction in the Argonaute protein were reported as follows.

## Results

In following contents, we will first comment in detail about binding modes obtained by docking, the structural stability, flexibility and motion correlations observed in the simulations of the three systems of free Ago, Ago-miRNA and Ago-miRNA-mRNA complexes, and then discuss the hydration effects and H-bonding network in Ago binding channel. After this, a detailed discussion of the thermodynamic analysis is presented.

### Binding models of miRNA, mRNA to Ago

The binding model of miRNA to Ago is extremely similar to that of DNA to Ago in the crystal structures (3F73, 3DLB). The nucleotides 1 and 2 at the 5′-end of miRNA are anchored within the binding pocket in the Mid domain, with their phosphate (base) oxygens (nitrogens) hydrogen-bonded to the side chains of highly conserved residues (Val678, Asn445, His441 and Arg 442) as previously observed in the *Thermus thermophilus* argonaute with 21-base DNA complex (Supporting Information Figure S1 (I) and supporting Dataset S1) [20]. The magnesium 681 is coordinated to the first and third phosphates from the 5′ end, as well as the carboxy-terminal carboxylate end (Val678) of the PIWI domain (Figure S1 (II) and supporting Dataset S1), and the second magnesium 679 is also well located in the catalytic triad (Figure S1 (III), supporting Dataset S1) [20], [21]. Nucleotides 22 and 23 at the 3′ end of the miRNA are anchored within the binding pocket in the PAZ domain (Figure S1 (IV) and (V)), with the oxygens of the phosphate linking base 22 and Arg228, and three H-bonds formed with Trp239, Glu206, Pro208 (Figure S1 (IV) and supporting Dataset S1). And the 3-OH of nucleotide 23 hydrogen-bonded to acidic Ser218 side chain, as previously observed in PAZ–siRNA [20], [21] and PAZ–single stranded RNA complexes (Figure S1 (IV) and supporting Dataset S1) [20], [21]. In addition, there are extensive hydrogen bonding and salt bridge formations between the backbone phosphates of the miRNA strand and the nucleic-acid-binding channel in the protein (Dataset S1) and proposed models of the catalytic cycle [20], [21].

In the ternary system, the bound mRNA strand threads its way within a central channel in the Mid and PIWI-containing (Mid and PIWI) lobes of the bilobal scaffold of Ago, with its segments 2–8 (seed) hydrogen-bonded to miRNA (Figures S1 (I) and (V)). The bases are splayed apart at the nucleotides 1–2 from 3′ end, with base 1 stretched over the plane of the binding site and the last 8 nts stretched out from the PIWI groove which is almost perpendicular to the miRNA line.

These results suggest that our binary and ternary complex models derived from docking are reliable.

### Dynamics and stability of overall protein structures

For all three systems, the temperature, total energy, mass density, and volume are found relatively stable throughout the MD simulations. The conformational drift of Ago structure is also measured in terms of the root-mean-square deviation (RMSD) with respect to the starting structure (supporting Figure S2). Plotting the RMSD of all Cα atoms as a function of time for the three simulation copies reveals relatively small changes in the structure, indicative of satisfactory simulations performed (Figure S2).

The RMSD values (Figure S2) observed for Ago show a relatively wider range (4.15±0.63 Å) in the stable time, suggesting that significant domain movements are involved. Whereas the RMSDs of Ago bound with guide RNA vary within a much narrower window of 2.72±0.31 Å and, in all cases, appear more stable, apparently due to the presence of the binding to miRNA. Notably, for the ternary complex, the RMSDs observed restore to a wider range (2.80±0.52 Å) similar to that of the free Ago, suggesting that this complex possesses more fluctuations after binding to the second RNA (mRNA). Actually, this increased structural flexibility of the ternary system is attributed to the existence of more flexible subdomains such as the loop 1 (amino acids: 198–239) and sheet 1 (amino acids: 74–102) (see following section E of **Results**), in accordance with earlier observations [21], [22]. The visual analysis of each trajectory obtained from these three simulations also supports above observations. These phenomena imply that for Ago, its binding to the guide RNA greatly increases the complex's stability, yet when the binary complex further binds to another target RNA, the stability of the complex is slightly decreased due to the perturbation of the free 5′ end of mRNA (see more discussion below).

**Figure 1:**
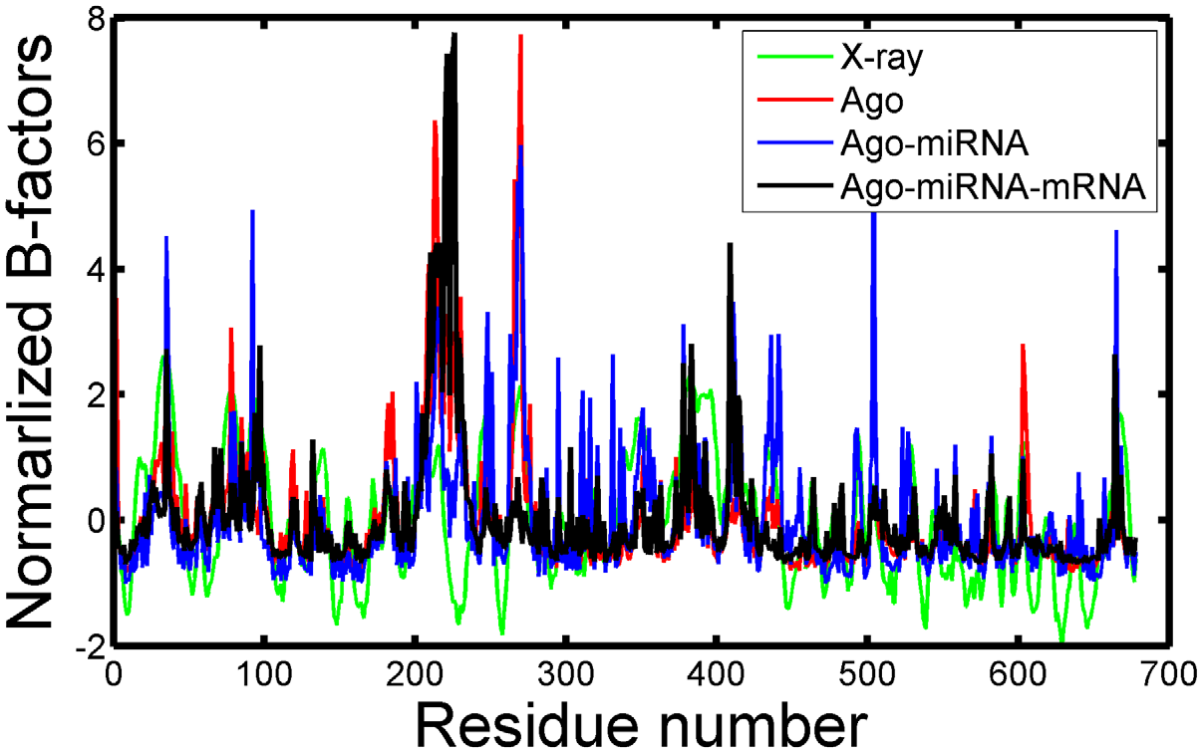
The normalized B-factors for free Ago, Ago binary and ternary complexes together with the X-ray structure (3F73.pdb^20^).

The B-value [25] calculated from all-atom MD simulation provides another approach to evaluate the convergence of the dynamical properties of the system. In the present work, the obtained normalized B-value [25] results (Figure 1) well agree with those reported in the X-ray structure (3F73.pdb [20]). In particular, the simulations reproduced the sharp peaks observed in the crystallographic structure around loop 1 and sheet 1 (supporting Figure S3). In addition, all those residues with higher fluctuation values are found belonging to the highly mobile solvent-exposed amino acids (loop 1 and sheet 1) in the PAZ domain. On the contrary, only a small degree of flexibility is observed at the Mid domain (326–462). As to the PIWI domain (amino acids: 463–678), it also exhibits much larger stability than the PAZ domain, which is consistent with its structural role in the heterotrimer [20].

To extend this analysis, a principal components analysis [26] was performed on the equilibrated portion of relevant trajectories, ending in results agreeing well with the B-value analysis in that the principal component contains the motion of loop 1 and sheet 1 region. And this region appears to be significant and be modulated by the presence of bound miRNA within the central cavity. This consistency between the simulation results and experimental observations proves the reasonability and validity of the models.

### Motions of PAZ, Mid and PIWI domains

**Figure 2:**
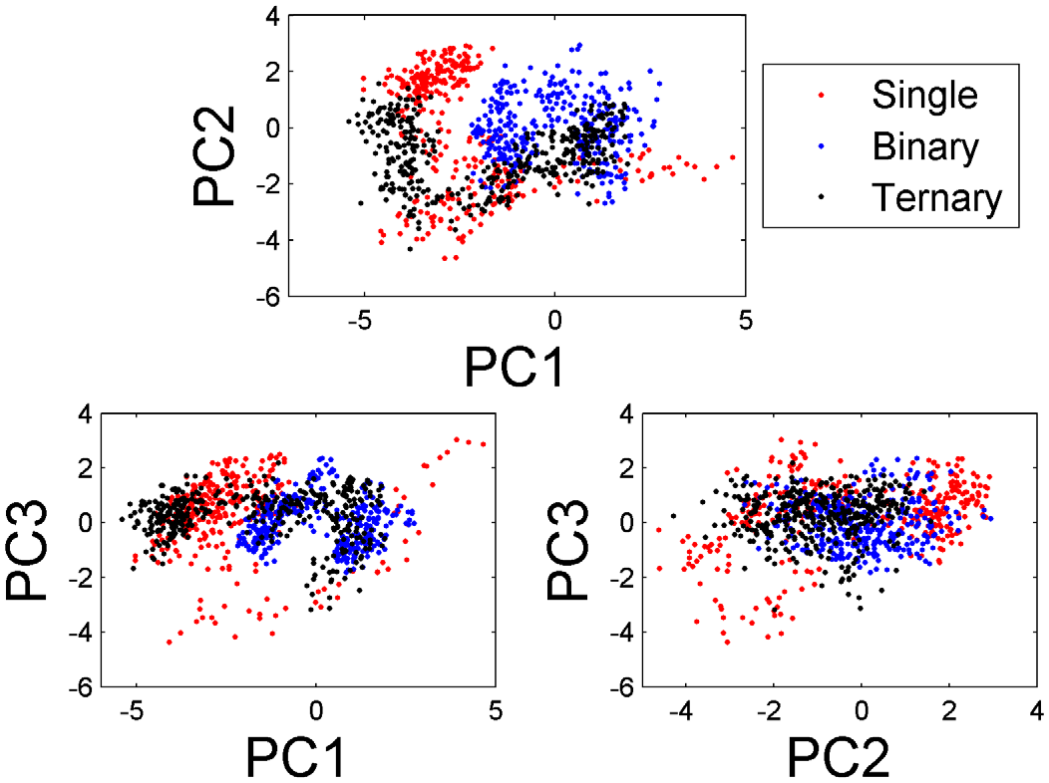
Charts of the first three principal components (PC1, PC2, and PC3) calculated from the 3000 structures of the three MD trajectories of the free Ago (red), Ago-miRNA complex (blue) and Ago-miRNA-mRNA complex (black).

Principal component analysis provides one way of extracting large-scale motions in proteins. Similar in spirit to a normal-mode analysis, PCA breaks up the total motion into contributions, each with a pattern of coherent motion [26]. In this study, the simulation trajectories of each system were analyzed for dominant collective displacements using PCA of the fluctuations of Cα atoms [27]. The first two eigenvectors in the ternary model from the PCA capture about 55% of the variance of entire crystallographic ensemble and thus represent large-scale collective motions, with subsequent eigenvectors capturing significantly less fluctuations (see Supporting tables (Table S1)). It is thought that the large displacements seen in the first few eigenvectors of such analyses represent functionally important global movements, while lower-order eigenvectors are smaller, localized fluctuations that do not influence the function [27]. Accordingly, PCA studies focus on these dominant motions and Figure 2 shows the projection of each member of the crystallographic ensemble onto the plane defined by the top two eigenvectors.

On these projections, clusters of stable states were observed with two apparent features from these plots. Firstly, the clusters are well defined in all three systems, indicating that these systems sample two (for the single and ternary systems) or three (for the binary system) distinct minima during the molecular dynamics trajectory. Secondly, the single system is found covering the largest region of the phase space, the ternary system covers the second largest region and the binary system covers the smallest area. By analyzing the structural clusters, the significant shift seen between free Ago and Ago ternary complex clusters is found mainly resulted from large conformational change in PAZ domain (see more discussion below). Our observation thus corroborates with the idea that free Ago has higher flexibility than the bound Ago with RNAs at room temperature.

To characterize the collective motions represented by the dominant eigenvectors, interpolations between the extreme projections of the crystal structures are provided in section E (in the results), with purpose to convenient compare with following results of section D.

### Conformational changes of RNAs in Ago complexes

**Figure 3:**
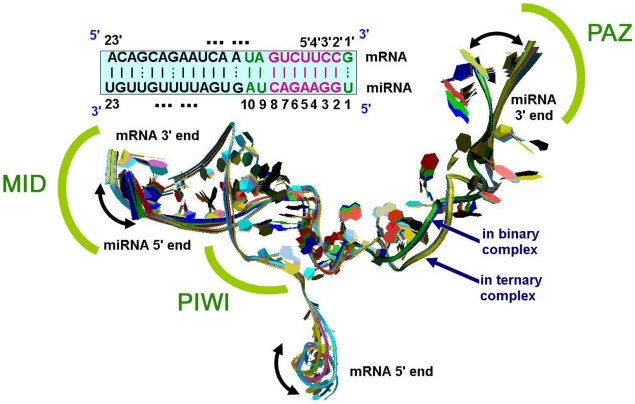
Superposition of the structures of miRNA and its target from the binary and ternary complexes respectively. Binary complex: two extreme projections of miRNA on PC1 (blue and red) and average structure (green); Ternary complex: two extreme structures of miRNA-mRNA on PC1 (cyan, yellow), and average structure (pink).

In order to reveal a clear-cut structural difference of the RNAs in Ago binary and ternary complexes, PCA was also performed on each trajectory of the two complexes independently. The top three eigenvalues for miRNA in the binary system explain 56.8% of the total variance, and 70.1% of that in the ternary system (Supporting tables (Table S2)). As for mRNA, in the ternary system 81.3% of variance is represented by the first three dominant eigenvectors (Supporting tables (Table S3)). Figure 3 shows the superposition of extreme projections on PC1 of structures of the guide RNA and its target. The bulk of the displacement is seen in the 5′-end, involving five nucleotides in mRNA, and eigenvector 1 represents a concerted swing event in the solvent environment.

For miRNA, no evident conformational variations are found, which is shown by the good superposition of the extreme structures in the binary system, and the same story holds for the ternary system (Figure 3). However, the comparison of the conformations of miRNA between the binary and ternary complexes shows a significant change in segments 15–23 from 5′-end. This part of miRNA rotates in a clockwise direction up to 50° and translates ∼5 Å from the binary to ternary complexes in the presence of its target, representing the most open state of the cleft (PAZ binding pocket). Widening of the PAZ-PIWI distance in the substrate-bound forms represents the accommodation of nucleic acid into the RNA binding channel. Eigenvector 2 represents a significantly smaller displacement of atoms and shows quite similar results as PC1 and is thus not discussed here for space saving.

Clearly, the present miRNA-target exhibits good pairing to the 5′ end of the miRNA as shown in Figure 3 (upper part), no evident interaction is found for base-pairing to the 5′-end of the mRNA with its guide RNA (see supporting Figure S4 and Dataset S2), failing to meet the demand of a canonical site rule, i.e., it has enhanced 3′ pairing in addition to a sufficient 5′ seed [28]. Therefore, the functional importance of the 5′ end of mRNA might be related to other physical characteristics or functions such as the dynamic properties in the binding complex. We could speculate that, due to the violent sway of 5′-end hinged by nucleotide 18, the cleaved part of mRNA can be easily disassociated from the Ago system, thus accelerating the catalysis cycle of miRNA.

### Correlation between RNA conformational change and motions of Ago motifs

**Figure 4:**
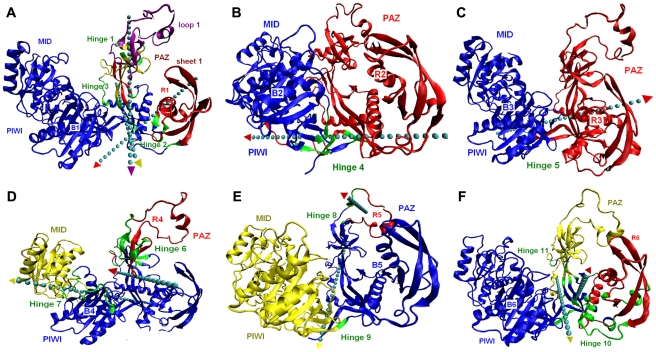
Principle-component mode of domain movement. The first and second motion modes of Ago in single system (A–B), binary system (C–D) and ternary system (E–F). Arrows represent the axes of motion. The colors of the arrow shaft and head correspond to the color of the static and dynamic domains, respectively. Hinge residues are colored green, fixed domains are in blue, moving domains are in red, purple and yellow.

As the RNA binding channel of Ago consists of several conserved motifs, the Ago conformational switch probably induces the movements of the bound RNAs. To reveal the correlations, the motion modes of Ago in each structure projected in PC1 and PC2 identified by DYNDOM [29] are presented and shown in Figure 4. PCA results show that the dynamics of the single, binary and ternary systems appear to occur mainly along the first and second principal components, the subspace of which accounts for, 57.9%, 34.6% and 54.4% of the overall motion, respectively (Supporting tables (Table S1)).

As for free Ago, DYNDOM analysis discovered two key amino acid segments related to the motion of the first principle component, viz., loop 1 and sheet 1, two highly conserved subdomains in PAZ domain (Figure 4A). Loop 1 shows an independent movement of helical twist by hinge 1, and sheet 1 exhibits as a dominant contributor among the residues involved in the interdomain rotation and bending. And the large scale motions of loop 1 are also captured by PC2 with respect to domain R2 (Figure 4B). These results indicate the existence of a reasonable agreement between the X-ray sub-domains (3F73, 3DLB) and those in the PCA eigenvectors, particularly those in eigenvectors 1 and 2. Therefore, we speculate that the large scale motions of PAZ play a mechanical role for the Ago binding activity, resulting in the generation of a wider and shorter nucleic acid binding channel necessary to hold and orient the bound RNA guide strand.

In Ago binary complex, the eigenvector explaining the largest part for the variance of the trajectory of Ago corresponds to the deformation in which a conformation containing two large subdomains (the blue, B3, and red, R3 in Figure 4C) forming a crescent-shaped base. In the binary complex, the first eigenvector represents a deformation which opens and closes the boat-like shape of the nucleic acid binding channel (Figure 4C), connected by a stalk-like linker region between the N-terminal and the PAZ domains, an interdomain connector cradles the structure. The second eigenvector displays a pivotal rotation of the subdomain loop 1, and the conformational changes are extended to the base PIWI-containing lobes (Figure 4D, with detailed residue numbers seen in Supporting tables (Table S4)). Clearly, this subdomain movement of the protein effectively adjusts the RNA positioning and, in this way, avoids the clashes with other parts of the full-length protein, which also provides a correct position for the binding to target RNA.

In mechanistic terms, we favor the view that the conformational changes in domains B3 and R3 (Figure 4C) and associated sliding and flipping of the miRNA-strand (Figure 3) are triggered by widening of the substrate-binding channel between the PIWI and N domains to accommodate a lengthening of the target RNA complex. Such changes not only push the PAZ domain away but also release the 3′ end of guide strand from the PAZ-binding pocket (Figure 4C). During our simulation, in most of the cases, the simple stretching and twist movements for such large subdomains (half of the protein) of Ago display fewer fluctuations of atomic coordinates. This is well consistent with the RMSD plot of the binary complex, which is the most stable one among the three systems. It is likely that the first and second motion modes for PAZ domain in miRNA-Ago complex are related to the miRNA 3′ end conformational switch, because the fluctuations of the eigenvalues corresponding to these two components are in good accordance with the conformational change profile of miRNA (Figure 3 and Supporting tables (Table S1)).

In the ternary system (Figure 4, E and F), the first two animated eigenvectors of Ago correspond to the stretching motion of a helical fragment around loop 1 in PAZ domain, which is similar to the previous one in free Ago, except for the immobilization of sheet 1 relative to PIWI domain. Half of the residues (loop 1) in this case have more pronounced contributions to the fluctuations of the atomic coordinates. In addition, Ago also shows a twist movement around the linker region of the protein (Hinges 10 and 11), which might explain why miRNA shows different conformations in the binary and ternary systems (Figure 3).

Thus, we conclude that the first and second components may truly correlate with the conformational dynamics of miRNA in the binding channel of Ago. No motifs are found to be likely related to the target RNA conformational change, which are in good accordance with the conformational change profile of mRNA (Figure 3). The comparison of the PCA results of free Ago and Ago ternary complex further reveals that the first component of free loop 1 is highly similar to that of loop 1-miRNA (Figures 4, A and D), suggesting that it is a fundamental motion of loop 1.

The extreme conformations of PC1-PC2 are further used as input to detect the motion hinges [30] as well as to quantitatively analyze the magnitude of the protein movement. Table 1 shows the hinge parameters for the first two PCs, and each hinge is indicated in Figure 4, A–F. From this table, we can find that hinges 1 and 8 (Figure 4, A and E) have the largest magnitude of rotation of about 25° in all hinges, making the PAZ domain partially open in the single and ternary complexes; And the biggest closure movement is subjected to loop 1 and sheet 1, indicated by hinges 6 and 10 of ∼100% closure degree in axis direction. The complicated and large movements of PAZ domain around these hinges imply its functional importance. In the binary system, the relative opening-closure degree of two subdomains (B3 and R3) is represented by hinges 5, 6 and 7, showing a trend of lengthening and widening of the nucleotide-binding channel (Figure 4). In addition, another interesting finding is that five out of eleven hinges (1, 3, 6, 8 and 11) are relevant to loop 1, the main part of Ago, which might further demonstrates the predominant role of loop 1 in Ago for the RNA binding.

**Table 1:** Hinge parameters for the first two principal components (PC1-PC2) of Ago.

		PC1			PC2		
Species	Hinge	Rotation angle (°)	Translation (Å)	Closure* (%)	Rotation angle (°)	Translation (Å)	Closure* (%)
Single	1	25.6	−0.4	84.4			
	2	14.5	−0.5	52.4			
	3	20.5	−0.9	68.5			
	4				19.3	−0.2	6.8
Binary	5	11.9	0.4	19.6			
	6				11.3	−0.5	94.2
	7				12.0	0.6	35.2
Ternary	8	24.9	1.5	82.6			
	9	12.5	0.7	82.4			
	10				12.9	0.1	99.7
	11				8.9	0.1	84.3

For each hinge, the effective rotation angle, the translation distance and closure degree between extreme conformations of the moving domain were reported.

*A percentage measure of the degree of closure motion defined from the square of the projection on the closure axis. The detailed residue numbers of each system are presented in Fig. 4.

**Figure 5:**
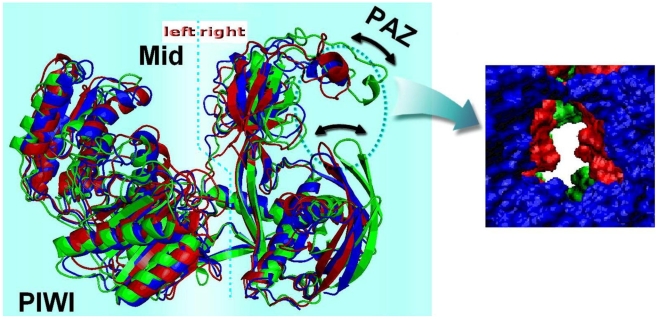
Superposition of the average structures from each MD trajectory and right cross-section for PAZ domain. Free Ago and Ago in binary and ternary systems are shown in blue, red and green colors, and their responding PAZ entrance areas are 396, 299 and 274 Å^2^, respectively. The cross-section area of RNA is 95.0 Å^2^.

In addition, to compare the conformational changes in different systems, the average geometries of Ago from each of the simulations are structurally superimposed (Figure 5). It can be seen that in free Ago (blue), the PAZ domain approaches most to the geometric center of left subdomain (amino acids: 310–678) (Figure 5 left), with a distance of 33.4 Å between the two geometric centers of the Cα atoms of left and right subdomains (amino acids: 1–310) and an entrance area of 396 Å^2^ (right cross-section) of PAZ domain (Figure 5 right, in blue color). After binding to a miRNA, this nucleic-acid-binding channel between the PAZ- and PIWI-containing lobes of argonaute is slightly lengthened to 34.9 Å, but the entrance significantly shrinks to ∼299.4 Å^2^ (Figure 5 right, red color). And the channel can be further greatly lengthened up to 41.0 Å after binding to another target RNA strand, with the PAZ entrance being ∼25.4 Å^2^ more narrowed compared to the binary system (Figure 5 right, green color). From the above conformation studies on the three Ago structures, an unexpected mechanistic insight emerges that the short and wide binding channel in the free state is able to relieve the topological stress of Ago on the guide RNA, and make the RNA 5′-head easily attach to the 5′-phosphate-recognizing Mid binding pocket from the entrance of PAZ domain [20], [31].

### Conformations of seed

In order to understand the importance of base paring on the stability and flexibility of the seed (bases 2–8), the all-atom RMSDs of the seed bases of miRNA in free and bound states are evaluated (supporting Figure S5). It is observed that the segment in unbound state undergoes larger fluctuations, increased up to 6 times compared to that in the state bound to a target RNA (RMSD = ∼1.5 Å, see supporting Figure S5). The 2–8 segments of the bound guide miRNA form a stacked array, such that the solvent-exposed Watson–Crick edges of bases 2 to 8 in the ‘seed’ segment are positioned for nucleation with target RNA [20]. Therefore, we conclude that large fluctuations of unbound bases should be of functional significance, which could increase the possibility of formation of the seed duplex by base-paring H-bonding interactions.

**Figure 6:**
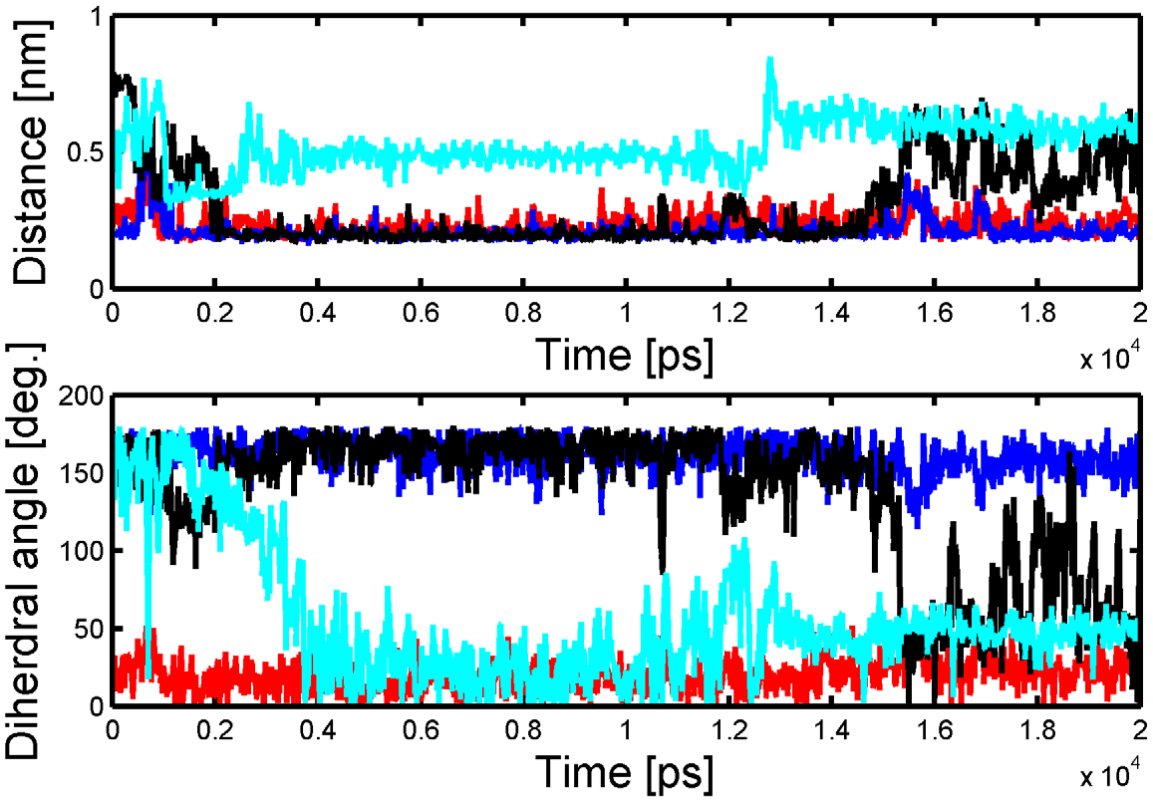
Hydrogen bond distances and diherdral angles of base pairs of 7-7′(red); 8-8′(cyan); 9-9′(blue), 10-10′(black). The dihedral angles are A7:C2-N1-U7′:N3-C2; C8:C2-N3-G8′:C2-N1; U9:C2-N3-A9′:N6-C6; A10:C2-N1-U10′:N3-C4, representing the base-base angles between two base pairs.

In addition, the conformational changes of two bases 9 and 10 proximal to the seed segments, which might be crucial for the catalytic cleavage of target RNA by PIWI domain, are also analyzed [20]. Figure 6 shows the RMSDs of distances of four inter-base-pair-hydrogen bonds and dihedral angles (base–base ring) for A7-U7′, C8-G8′, U9-A9′ and A10-U′10 versus simulation time. As expected, the distances of A7-U7′ and C8-G8′ (seed) are less than 3.0 Å over the entire trajectory, indicating that the structure of the two-base-pair dimmer is stable. Interestingly, for U9-A9′, in the fist 0.2 ns, H-bonds are formed and subsequently break and the distance between two corresponding bases increases up to 7.5 Å until to 20 ns, afterward, this distance descends to ∼3.0 Å and the H-bonds form again, but at 15 ns, the H-bonds break again and remain broken until to the end. This result reveals that nucleotide 9 may switch between two kinds of conformations in the binding site (Figure 3), which might explain why there is a 7- or 8-mer seed match in different species, except for the conserved 6 mers [4], [32].

During the whole simulation process, nucleotides 10-10′ are found undergoing larger conformational changes. The H-bond distance remains below ∼3.0 Å during the first 1.0 ns, and reaches ∼6.5 Å in the following 3.0 ns. Afterward, it descends but still remains large (5.0–6.0 Å). The dihedral angle profile describing the base-base rings of the four base-pairs exactly matches the H-bonding profiles (Figure 6), in that the base-pair dihedral angles for rotation around the A7:C2-N1-U7′:N3-C2 (abbreviated as T1) and C8:C2-N3-G8′:C2-N1 (T2) bonds are planar and oscillate around 20° or −20°, being close to the optimal geometry of isolated seed segment. These data indicate that the hydrogen bonds formed between 7-7′, 8-8′ base pairs are stable (3.0 Å). The U9:C2-N3-A9′:N6-C6 (T3) bonds show the same pattern as T1 and T2 from the beginning to 15 ns, but deviates for the last 5 ns, further supporting the switch of conformations of U9 in the whole trajectory. Different from above T1 and T2, the dihedral angle for 10-10′ (T4) remains about 170° in the first 2 ns, and then is inverted to 120° for most of the last trajectory, leading to the disruption of the initial H-bonding. This computed binding mode is identical to the local geometry reported in the crystal structure (3F73), i.e., the base 10-10′ equilibrates to a departure conformation, which makes the phosphate diester bond of U10′ approach the catalytic site in PIWI domain, so that the cleavage event can take place (see more discussion in section G). These findings might also explain, from an atomic level, why the repression of gene expression was not affected by a mismatch at position 10 for miRNA and target [28], since the two bases 10-10′ do not have to be complementary and cemented together for target recognition.

**Figure 7:**
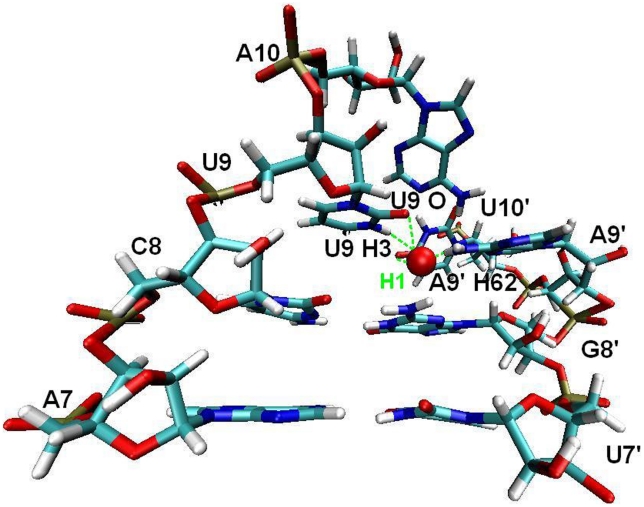
One-water-mediated-hydrogen bond networks for base pairs (7–10). Green dashed lines represent the hydrogen bond.

The X-ray crystal structure of Ago complex indicated that an array of water molecules play an important role for the receptor-mRNA binding (3F73, 3DLB, 3HM9). To study the specific roles of water molecules in the binding of mRNA to Ago complex and in the conformational change of seed site, we further performed a detailed analysis on the MD trajectory of the ternary complex using the VMD program [33]. Figure 7 shows one representative snapshot isolated from the MD trajectory.

By dynamic simulation of the ternary system, it is revealed that the hydration around A7-U7′ and C8-G8′ is not defined, while the baseparing of U9-A9′ and A10-U10′ is complemented by a highly specific hydration site occupied by one water molecule. Because of poor accessibility, this site is vacant during the initial 7 ns of simulation, but at 7.0 ns into the simulation it becomes hydrated and remains occupied for the rest of the simulation. And this hydration site forms five H-bonds with U9, A9′ and A10 as shown in Figure 7. This observation confirms the above H-bonding picture for A10-U10′, where after 7.0 ns the distance of the two bases becomes stablized and enlarged to 6.0 Å (Figure 6), thus a relatively deep pocket between the two bases is formed to accommodate a water molecule. This hydration site shows residence time of the water molecules in a long range of 15 ns. Therefore we speculate that the long-residing and presumably tightly bound water molecule contributes considerably to the stability of U10′ in the catalytic binding site in PIWI domain.

### Hydration and ion interaction at PIWI catalytic site

In the crystal structure of Ago complex (3F73), a well-ordered divalent metal ion (Mg^2+^) is bound to the C-terminal carboxyl groups of triad D474, D541 and D653 at the PIWI domain [20]. In order to investigate the physical function of this ion in the PIWI active site of the ternary complex, possible hydration effects and ion interactions were investigated.

**Figure 8:**
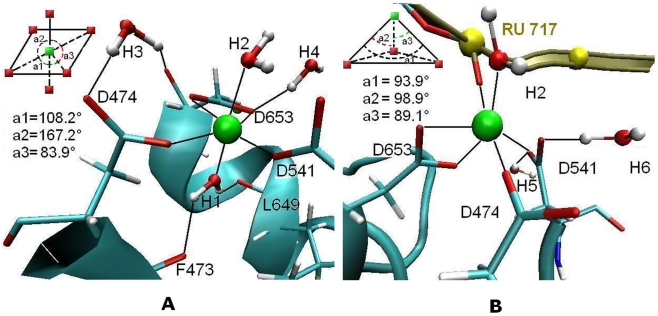
Equilibrated coordination of the active site magnesium ions 679. (A) binary 679. H1 and H2 keep constant; (B) ternary 679. H2, H5 and H6 keep stable. All other water molecules do not keep constant in the place. a1 (D474–D541), a2 (D541–D653) and a3 (D474–D653) are the angles between these three residues.

An analysis of the water density around the PIWI catalytic site reveals several highly occupied hydration sites (Figure 8). Six hydration sites are observed in the binding pocket near the Mg^2+^ in both binary and ternary complexes referenced as H1–H6 in the following text. In the binary complex, three of these sites (H2, H3 and H4) are above a plane formed by the triad carboxyl groups, with first site (H1) below this plane interacting with the two O (F473) and O (L649) atoms (Figure 8). With the abolishment of hydration sites H1, H3 (D474) and H4 (D653) in the ternary structure, two new hydrations sites H5 and H6 are formed by OD2 (D474) and OD1 (D541), working as glue to stabilize the catalytic center of the tetrahedron structure. These hydration sites appear to compensate for the crystal waters in 3F73 [20], in which only sixteen irrelevant water molecules were crystallized. Hydration sites show residence time of individual water molecules in the long range of 0.1–20 ns except H3 and H4 which occupancy is only around 50% of the entire trajectory (see Supporting tables (Table S5)). The long-residing and tightly bound water molecule (Table 2) in H2 is functionally significant, facilitating the RNA hydrolysis during catalytic cleavage by RNase-H-containing nucleases together with the Mg^2+^ cation [34], although experimentally, at present it is not possible to identify the position of the water molecule that would participate in and be positioned for in-line attack on the scissile phosphate target.

**Table 2:** Active site interactions, including protein-Mg^2+^ interactions, protein-water interactions and interactions with the Mg^2+^-coordinated water molecules.^a^

	Length (Å) [standard deviation Å]		
	Ternary	Binary	X-ray
D474 OD2-Mg^2+^	1.89(0.04)	1.86(0.04)	3.21
D541 OD1-Mg^2+^	1.88(0.04)	1.87(0.04)	2.35
D653 OD1-Mg^2+^	1.93(0.06)	1.96(0.06)	2.99
D653 OD2-Mg^2+^	1.97(0.07)	1.94(0.05)	2.56
U717 O1P-Mg^2+^	1.89(0.05)	-	-
H1-Mg^2+^	-	2.05(0.07)	-
H2-Mg^2+^	2.03(0.07)	2.04(0.06)	-
D474 OD1-H3	-	3.01(0.08)	-
D653 OD2-H3	-	2.85(0.05)	-
D653 OD1-H4	-	2.98(0.07)	-
D541 OD1-H5	2.79(0.83)	-	-
D541 OD2-H6	4.23(1.26)	-	-
U717 P-H2	3.63(0.17)	-	-
D653 OD1- D474 OD2	2.76(0.81)	-	-
D474 OD2- D541 OD1	2.72(0.73)	3.05(0.88)	-
D541 OD1-D653 OD1	2.87(0.67)	2.96(0.71)	-
D653 OD2- D474 OD2	-	2.66(0.64)	-
H1-F473 O	-	2.47(0.69)	-
H1-L649 O	-	2.85(0.77)	-

a The simulation results were calculated over the last 6 ns with data collected every 1 ps. ^-^ not available or not necessarily measured. Shown are average values and standard deviations in the parentheses.

Interestingly, the transition from binary to ternary binding mode of the system does not cause any water switch in H2 but results in the vanish of H1. In the binary structure, as schematized by the sketch map on Figure 8A, the four oxygen atoms OD2 (D474), OD1 (D541) and OD1, OD2 (D653) form a ‘strict’ plane, indicating by the sum of angles between the triad residues of 359.3°. Mg^2+^ is well located at the center of the plane of the four-membered ring. However, it is likely that the planar structure is rather unstable due to the short stacking distance and effect of oxygen–oxygen electronic repulsion. Previous work has found that the metal ion bound to the C terminus observed in both the RNA-bound and free forms of AfPiwi was not observed in PfAgo, even when crystals were soaked with Mn^2+^, the possible reason is that the single metal ion can not keep stabilized in the plane without additional help from outside of the triad [19]. Based on this thought as well as the simulation results, we then found out the particular significance of the well circumscribed hydration site H1. The water molecule occupying this site stabilizes the planar state of the Mg^2+^ ion mediated-tetramer by a strong water-ion electrostatic attraction, with its two hydrogen atoms hydrogen-bonded to the two adjacent carbonyl groups of F473 (H-bond distance is 2.29 Å) and L649 (2.32 Å) utilizing their acceptor abilities, respectively. The stability is confirmed by the RMSD plot of the Mg^2+^ with interfaces 7 atoms (Mg^2+^, four O atoms, and two water molecules at H1 and H2 sites) in the binary structure (Figure 8A), with an average RMSD less than 1.5 Å (see supporting Figure S6). In addition, the results of distances between these atoms are in the range of 1.8 to 3.1 Å (Table 2), also supporting the conclusion that the H-bonds in this system are stable.

However, this geometry is destroyed after the binding of a target RNA. Due to the attraction from O1(P)-RU717 on Mg^2+^ (Figure 8B), the basal plane is drawn away from the axial direction for 3.0 Å, and the metal ion is further pulled out of the DDD-constructed plane for 1.2 Å. This results in a regular tetrahedron structure (symmetrical) with the ion as the vertex, as revealed by the three similar dihedral angles in size (∼90°) (Figure 8B). The RMSD plot for this structure in entire trajectory (<3 Å, see supporting Figure S6) and the key distances between the involved atoms (Table 2) also demonstrate that this catalytic center in PIWI domain is quite rigid. Clearly, the movement of Mg^2+^ occurring upon binding of the target strand could avoid the clashes with other parts of the protein and bring the target RNA in correct position for cleavage. This finding also explains why the H1 is absent in the ternary structure, for: 1) no stable H-bond can be formed as in the binary complex because the Mg^2+^ is drawn too far from the two residues F473 and L649; 2) The pocket under the triad plane built by F473 and L649 becomes so much narrower in the ternary complex that no water molecule can easily enter.

### mRNA H-bond network

In addition to the H-bond network for miRNA-mRNA (seed) discussed above, we also examined the hydrogen bonds between the mRNA and Ago protein. Examined 3000 snapshot structures for the last 12.0 ns, we identified direct hydrogen bonds. Base 1′ is splayed and hydrogen-bonded to O of F640 with the backbone phosphate group hydrogen-bonded to the side chain (2HH1, 2HH2) of K440. Base 1 is the only residue before step 9 (here, step 1 is referred to the 1^st^ base counted from the miRNA 5′ end, step 2 is the 2^nd^ base of miRNA 5′ end, and so on for others.) on the target strand that makes base-specific contact with the Ago scaffold, and this observation is consistent with the reported sorting of small RNAs in Ago complexes [20]. A helix from the Mid domain appears to separate the miRNA and its target strand at the 5′ end, which state of separation is stable in the entire simulation time shown by the dynamics simulations. This unpairing of the first putative guide-target ‘basepair’ is in agreement with the observation that base pairing at this position does not appear to be required for target recognition [35].

The guide DNA–target RNA seed duplex spans positions 2 to 8, with the scissile phosphate (10–11 steps) on the target strand positioned opposite to the catalytic residues (D474, D541 and D653) of the RNase Hfold of the PIWI domain [20]. For steps 11–23, the basepairing is disrupted, and the target RNA subsequently runs in an almost perpendicular trajectory to the guide RNA, stretching out from the crescent base (Figure 3 and supporting Figure S4).

**Figure 9:**
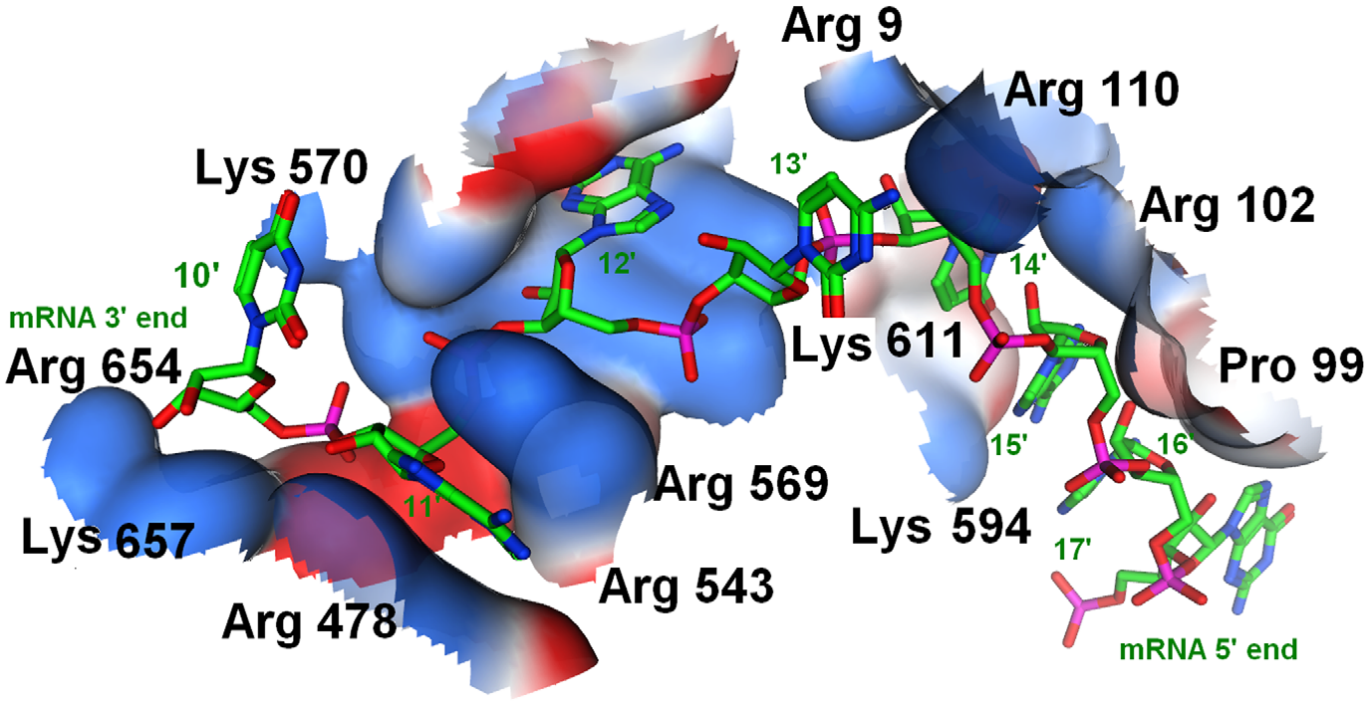
Electrostatic surface of the equilibrated structure model of mRNA-Ago. The electrostatic potentials were calculated by eF-site (http://ef-site.hgc.jp/eF-site/index.jsp). The key active site residues are shown in surface wall with the mRNA strand shown in ball and stick. Hydrogen bonds are formed between U10′-Lys657 (2 H-bonds) and Lys570 (2); A11′-Lys570 (2) and Arg543 (2); A12′-Arg569 (1); C13′-Lys611 (1); U14′-Arg102 (1). Only the surface of the key interacting amino acids is shown for the sake of clarity. The blue surface represents the basic area and red color for acidic area. Oxygen, phosphate, hydrogen, and nitrogen atoms are colored red, orange, green and blue, respectively. This architecture forms a large positively charged groove between the PIWI domain and the crescent base.

As seen from Figure 9, Ago binds directly to the middle-last part in the **GAAUCAAU** region of the sequence (5′-ACAGCA**GAAUCAAU**AGUCUUCCG). From segments 10–17, the plane of mRNA remains parallel to the wall of the groove and forms eleven H-bonds with seven amino acids in the ternary Ago complex ((U10′-Lys657 (2 H-bonds) and Lys570 (2); A11′-Lys570 (2) and Arg543 (2); A12′-Arg569 (1); C13′-Lys611(1); U14′-Arg102 (1)). This interaction is stabilized by the fit of the skeleton phosphate oxygen hydrogen-bonded to the guanidinium group of arginine and the ε-amino group of lysine along the narrow groove of PIWI. The six 5′ -end overhanging nucleotides (18–23) that are not adjacent to the binding channel freely stretch in the solution. Interestingly, we found that these key residues interacting with mRNA are all basic, building a positively charged channel for mRNA binding, as shown by the blue surface in Figure 9 (see detailed discussion in section **Thermodynamic Analysis**). This might compensate for the unavailability of the Ago/miRNA/mRNA complex, providing new information for exploring the interactions of the ternary complex in the mRNA binding channel since the bases of the target RNA can not be fully traced in the present X-ray structures (3F73.pdb, 3DLB.pdb).

### Thermodynamic analysis

As a complement to the structural data discussed above, presently the estimates of the binding free energy of the guide and target RNAs to Ago in various binding modes were performed. For space reasons, only the main points of our analysis were outlined below.

Table 3 shows the results of a typical set of MM/PBSA calculations for the binding of miRNA to Ago and mRNA to miRNA-Ago complex, where the five energy terms in Eqs. (3) and (4) in **Materials and Methods**, as well as the total binding energy and the experimental results are provided. It can be seen that the electrostatics, van der Waals, and nonpolar solvation terms are favorable, whereas the polar solvation and entropy terms are unfavorable for the binding in both two complexes.

**Table 3:** Binding free energy estimates for each model.^a^

Contribution	miRNA+Ago		mRNA + Ago–miRNA	
	Mean/(kcal·mol^−1^)	Std	Mean/(kcal·mol^−1^)	Std
Δ*E*_ele_	−10765.22	61.14	−1739.81	40.55
Δ*E*_vdw_	−330.00	11.10	−187.51	9.76
Δ*G*_np_	−48.51	0.18	−29.72	0.56
Δ*G*_pb_	11002.22	62.58	1844.13	35.63
Δ*E*_ele_ +Δ*G*_pb_	237.00	18.12	104.31	14.99
ΔH	−141.51	17.61	−112.91	11.26
*T* Δ*S*	−123.14	30.43	−82.99	25.53
Δ*G*_bind_	−18.37	8.01	−29.92	7.60
Experiment*	−13.67			

a All energies were estimated from a single trajectory calculating each component separately. Δ*G* represents the difference in energy between the complex and free subsystems, miRNA–Ago and mRNA–Ago+miRNA.

*Experimental binding free energies were calculated by Δ*G*_bind_ = *RT*ln(*K_d_*), where T is the temperature in K unit (300 K is used in this paper, R is ideal gas constant. *K_d_* = 0.1 nM).

Since the RNAs have a net negative charge, the energies are dominated by the electrostatics and polar solvation terms, which two energies can not even cancel, as can be seen in the fifth row from the bottom of Table 3. This is a manifestation of the dielectric screening of the solvent. Actually recently it has been argued that the net binding of RNA to protein is dominated by this term [36]. In addition, the van der Waals term is also rather large (−187 to −330 kcal/mol), correlating with the binding areas to protein of the molecule. But the nonpolar solvation term is small for the two molecules (−29 to −49 kcal/mol). As to the entropy term, it is intermediate in size (82–123 kcal/mol), for the entropy is dominated by the nearly constant translational (∼12%) and rotational contributions (∼13%), whereas the variation is caused by the vibrational contribution (∼75%). Concerning with the calculated changes in solute entropy, i.e., the *T*Δ*S*, they are physically reasonable, since the flexible RNA molecules often exhibit as large as even up to 360 kcal/mol entropy due to large vibrations [37]. In general, the total estimated binding free energy Δ*G*_bind_ fall in the profile of the observational results [38], [39]. However, from these results we have to confess that the standard deviations of each component of the MM-PBSA are relatively huge, which is caused by the big vibration other than the translation and rotation movements for such a big molecular system. Thus this propagation of errors might finally influence the accuracy of the total free energy obtained.

**Figure 10:**
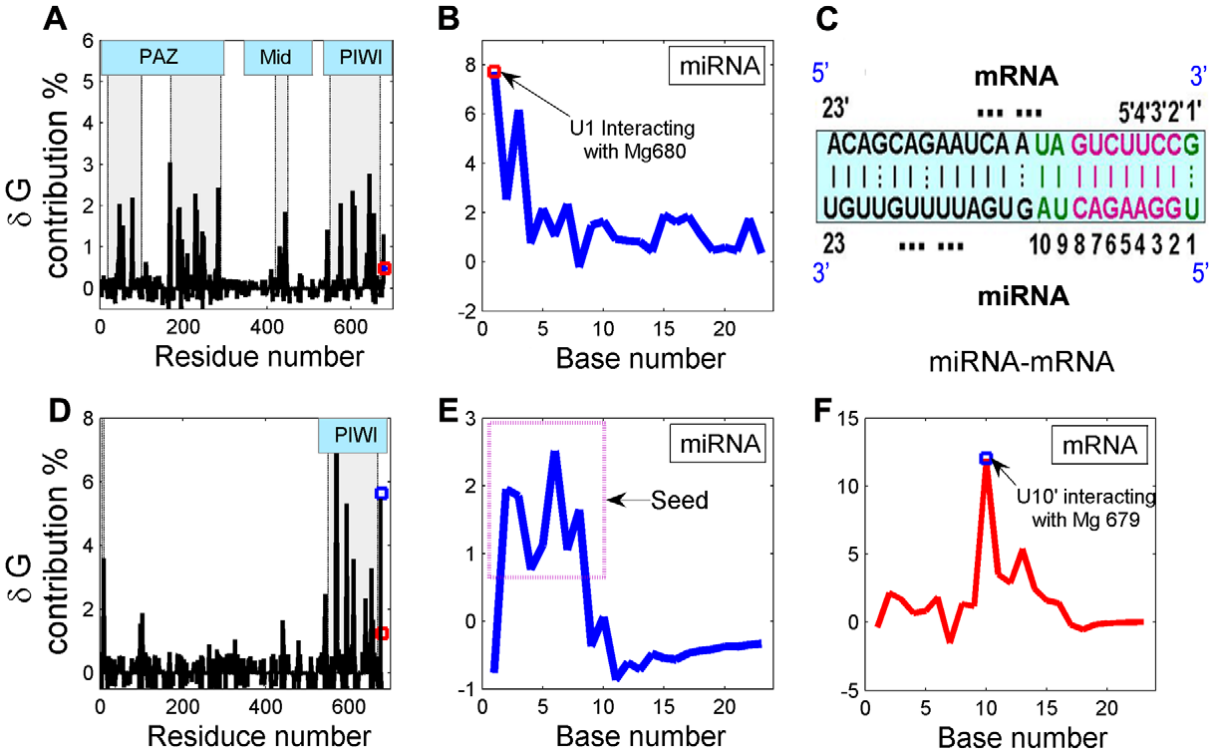
Thermodynamic analysis of the binary and ternary systems. (A) and (D): Contribution of each individual residue of Ago in the binary Ago-miRNA and ternary miRNA-mRNA-Ago systems to the binding free energy, respectively. (B) and (E): Contribution of each individual nt of miRNA in the binary and ternary systems to the binding free energy, respectively. (F): Contribution of each individual nt of mRNA in the ternary system to the binding free energy. The energy contributions of the residues correspond to the black solid lines, and those of miRNA and mRNA correspond to the red and blue lines, respectively. The gray boxes indicate the locations of the active site residues (binary: Arg47, Arg55, Arg77, Arg168, Arg188, Arg190, Arg228, Arg232, Arg282, Arg442, Arg543, Arg575, Arg604, Arg608, Arg644, Arg654 (16/80 in Ago); ternary: Arg9, Lys97, Arg102, Arg440, Arg543, Arg569, Lys570, Lys594, Lys611, Phe640, Arg654, Lys657).

In order to fully investigate the influence of the residues in protein on the interaction of binders, the RNA-residue interactions in each binary and ternary complexes were decomposed and compared systematically. Figure 10 shows the contribution of each residue and base to total Δ*G*_bind-miR_. For the miRNA binding to free Ago, the favorable residues can be classified into four clusters around residues R47, R228, R442 and R644, belonging to the PAZ, Mid and PIWI domains of Ago (Figure 10). Only those residues whose respective contribution to Δ*G*_bind-miR_ is above 1.4% are listed here, a threshold that ensures 80% of total Δ*G*_bind-miR_ is contributed by the selected residues. Very surprisingly, we find that the involved 16 amino acid residues (Arg47, Arg55, Arg77, Arg168, Arg188, Arg190, Arg228, Arg232, Arg282, Arg442, Arg543, Arg575, Arg604, Arg608, Arg644 and Arg654) are all arginine, which account for 20% in all 80 arginines in Ago protein (Figure 10). We also observe that there are extensive hydrogen bonding and salt bridge formations between the backbone phosphates of the guide RNA strand. As expected, these Arg side chains uniformly span the binding channel in PAZ and Mid domains but are more concentrated in the PIWI domain (6 arginine molecules) (Figure 10). This makes the catalytic site in PIWI domain more basic, thus facilitating the binding of a RNA guide sequence, which supports the previous assumption about the function of the RNA-binding groove [17].

After the discussion of contributions from amino acids, another important issue to address is to assess at what level basepairings contribute meaningfully to their function by thermodynamic analysis. Two most important bases are defined, i.e., 1^st^ and 3^rd^ nts from 5′ end, accounting for ∼15% of the total energy (Δ*G*_bind-miR_) (Figure 10). The first base of the guide RNA is flipped out and stacked with an Arg side chain, and the 5′-phosphate is placed in a highly conserved pocket in the Mid domain interacting with Val678, Asn445, His441 and Arg442 as previously observed in the *Thermus thermophilus* argonaute with 21-base DNA complex [20]. Interestingly, the 5′-phosphate is juxtaposed to the 3^rd^ phosphate, and the Mg^2+^ ion coordinated by the carboxylate group of a C-terminal valine or leucine stabilizes these phosphates [12], [20]. The distances between the two phosphate oxygen atoms and Mg^2+^ ion keep about 1.85 Å in all simulation time, indicating that the interactions between them are stable over time (Table 2), a result that is directly attributed to the effects of the metal ion which strengthens the protein-miRNA binding. This finding supports the idea that it is the 5′ phosphate that drags the whole miRNA strand and loads to the Mid domain to form a stable complex structure [40]. As compared to 5′-end, the 3′-end two nts show a low contribution (∼2%) to the total energy, which supports the experimental conclusion that the PAZ-RNA binding potential is weak [12].

**Figure 11:**
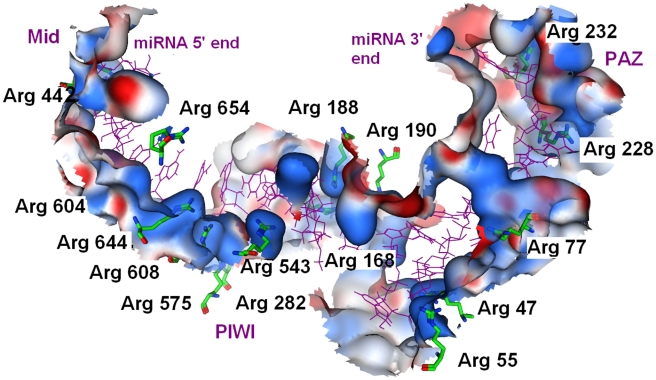
Equlibrated structure illustrating the close contacts between key active site residues (shown in surface wall) and the miRNA strand (shown in green stick). The blue surface indicates the basic area and red color for acidic area. This architecture forms a large positively charged groove between the PAZ domain and the crescent base, and a smaller one between the N-terminal and PIWI domains.

For the binding of mRNA to Ago-miRNA complex, from the analysis of Δ*G*_bind-mR_ 12 most influential amino acids, i.e., Arg9, Lys97, Arg102, Arg440, Arg543, Arg569, Lys570, Lys594, Lys611, Phe640, Arg654 and Lys657 were observed. These residues contribute 43.2% to the total Δ*G*_bind-mR_, with respective Δ*G*_bind_ contributions >1.5%. Arg9, Lys97 and Arg102 located in the PAZ domain are responsible for binding nucleotides 13–14, and the rest ones responsible for binding nucleotides 10–12 of the target RNA (Figure 9 and supporting Dataset S2). Once again and also interestingly, we find that all these residues are also basic arginine (6/12) or even more basic lysine (5/12), except one Phe640 (1/12), leading to the conclusion that the mRNA-binding-channel in Ago is also of basic feature (Figure 11).

Figure 10E shows the profile of contributions from each base of the miRNA in the ternary Ago-miRNA-mRNA to Δ*G*_bind-mR_. The 2–8 seed segments (in dotted line square) in miRNA totally contribute 7.1% to Δ*G*_bind-mR_, among which the 2^nd^, 3^rd^ and 6^th^ pairs (G∶C) are the most important yet the 4^th^ one (A∶U) is the least one, indicating the significance of G∶C base-pairs in the seed region. The contributions from other non-seed bases are sharply diminished, which, on the other hand, demonstrates the importance of the seed region for miRNA-RNA binding [35]. The experiment indicates that extensive 3′ pairing of up to 17 nucleotides in the absence of the minimal 5′ element (seed) is not sufficient to confer regulation. And it also supports previous suggestion that the mismatch at positions 1, 9, or 10 did not affect the target gene function, but any mismatch in positions 2 to 8 could reduce the magnitude of target regulation [28]. In summary, our data are consistent with a picture whereby the seed region is the primary interaction site, whose relatively short length renders it sensitive to single mismatches. As for mRNA, except for the seed segment, U10′ plays the most important role in the target RNA binding, which contributes 12.0% to the Δ*G*_bind-mR_ (Figure 10F). The significance of U10′ is revealed by the base-independent strong electronic attraction between its backbone phosphate oxygen and Mg^2+^ ion (Mg 679) in the PIWI domain (Figure 8B).

Overall, above observations provide compelling evidence that miRNAs recognize their targets mainly through limited base-pairing interactions between the 5′-end of miRNA and complementary sequences in the 3′ untranslated regions of the target mRNAs [41], [42], as well as through the interactions from amino acid residues in PIWI domain and the structural Mg^2+^ ion [20].

The energy relative contribution analysis for each amino acid and nucleotide was carried out based on the Generalized-Born decomposition approach, which is a major limitation of this work. Although in our work and many other cases, the net binding free energies are strongly correlated in the GB and PB models, a recent important work also found that the correlations of individual group contributions are highly variable that in some cases, strong correlation is seen, while in others, there is essentially none [43].

## Discussion

As mentioned above, argonaute is the core of the RISC complex involving in the posttranscriptional regulation of target gene [17]. In general, Ago consists of three (PAZ, Mid and PIWI) domains, where miRNA spans the PAZ-Mid and mRNA cleavage occurs in PIWI domain. In this study, by molecular modeling, dynamics simulations and thermodynamic analysis, we have obtained the dynamics process for binding of the miRNA and mRNA to Ago protein, as well the thermodynamic properties involved in the process, and thus at the atomic level gaining some insights into the principle of miRNA-target interactions.

To test whether the PAZ domain is of large structural flexibility and also to determine the function of this dynamics, we carried out dynamics simulations on the free Ago protein. The results show that the PAZ domain residues that undergo chemical shift changes are localized at the open end of the β-barrel and the appendage, which together form an elongated binding groove. And the function of the highly conserved loop and helix in PAZ domain is mainly related with their intrinsic nature of large and complex conformational changes in the free state [20], [44]. However, it appears that the fluctuations of these residues are less pronounced when a target RNA is present in the binding pocket, which is in agreement with the experimental observations [21] (see supporting Figure S3). The N-terminal and PAZ domains of large movements can therefore be compared to two lobster claws, while the relative rigid and static Mid and PIWI (RNase H) domains of Ago may be compared to the lobster tail and head. The PAZ domain movement is likely to be relevant to its function of ‘grasping’ the 3′ end of guide strand, since the large-amplitude motion is able to provide more chances for the guide RNA binding (Figure 4, A and B).

Once bound to a guide RNA, the domain movement of the protein and the adjustments of miRNA positioning become to be mainly responsible for widening the RNA binding channel, to avoid clashes with other parts of the full-length protein and to bring the further target RNA in correct position for cleavage, as revealed by the PC1 and PC2 captured motion of the protein in the binary complex (Figure 4, C and D).

In the ternary complex, the conformational transition in PAZ domain and associated sliding and flipping of the strand are triggered by widening of the substrate binding pocket for 3′-end of guide RNA. Such changes not only push the PAZ domain away but also release the 3′ end of the guide strand from the PAZ-binding pocket (Figure 4, E and F). Also, the atoms of PAZ domain exhibit less root-mean-square fluctuations (supporting Figure S3) in the target RNA bound-structure than in the target RNA-unbound one (Figure 1). These results are all directly linked to the observed release of guide 3′-end from the PAZ pocket [31]. The dynamic mode of the ternary model, particularly for the PAZ loop 1 supports a “two-state” mechanism in which the guide strand is anchored at both of its ends during the nucleation step of the target recognition, but its 3′-end is released from the PAZ pocket owing to topological constraints, after propagation of the duplex towards the 3′-end of the guide RNA [21], [40]. All these allow a molecular level understanding of aspects of the PAZ structural changes, and of the kinetic pathways of the intercalation and release process [21], [45].

For free Ago system where the simulation was started with water within the PAZ cavity (hydrophobic) [20], [46], the water was rapidly expelled and dropped to the level of ∼300 water molecules in the pocket (Figure 5), and further greatly reduced to ∼130 water molecules when the target RNA bound to Ago. The water occupancy of cavities in proteins shows a complex dependence on the cavity hydrophobicity and solvent conditions. This suggests that the hydrophobic lining of the cavity will favor partitioning of the hydrophobic molecules into the protein binding pocket. The hydrophobicity of 5′-end phosphate (CO[P]([O-])( = O)OC, logP = −4.36) is largely less than that of the 3′-OH end (OC1CCOC1, logP = −0.78) for a guide RNA, and this significant difference might explain why the PAZ domain preferentially recognizes siRNA 3′ end other than the 5′ end [45]. In addition, the present thermodynamic analysis also shows that the 3′-end nucleotide of the guide RNA contributes weakly (<0.5%) to the binding free energy (Δ*G*_bind-miR_) (Figure 10B) as compared with the most of other bases (>90% to total Δ*G*_bind-miR_), which is consistent with the observation that PAZ domain shows low-affinity of nucleic acid binding [12], [17].

The importance of the 5′-end phosphate of a guide RNA is revealed by its anchoring to the Mid domain through interactions with a divalent metal ion [17], thus resulting in ∼8% contribution to the total binding free energy (Δ*G*_bind-miR_) from 1^st^ nucleotide of the guide RNA. This finding might, from another aspect, explain why the PAZ domain does not preferentially bind to the 5′-end of a guide RNA.

Target selection in RNA silencing is governed by the “seed sequence” at the 5′ end of the guide strand, with important prerequisites for recognition including the guide-strand and target-sequence coexpression and accessibility of the target site [47]. Based on the dynamic analysis of the seed region, several interesting phenomena were found and analyzed. Firstly, the unpairing seed segment of miRNA is shown as flexible and dynamic, which can also be vividly compared to the lobster claws, and once the target is “grasped,” these “claws” become fixedly locked and the guide-target interaction is enhanced. Therefore, it is concluded that the seed itself is likely to function as a nucleation site for the miRNA–mRNA hybridization [48]. In addition, the formation of a nucleation anchor may be a prerequisite for the subsequent steps in complex assembly. It is also conceivable that preferential interaction at the seed region could be required to drive productive complex assembly. In this model, this complementarity produced interaction contributes about 7.1% to total mRNA binding free energy to Ago binary complex, indicating the nucleation effect is evident but might not be predominant for the target RNA recognition [28].

We now know that vast majority of animal miRNAs base-pair with imperfect complementarity with their mRNA targets. Although most bioinformatics approaches have been related with that a miRNA is perfect or near-perfect complementarity between the proximal (5′) region of the miRNA and mRNA, also known as the “seed” region or the “nucleus” (see reviews in ref [49]). Recent study has revealed synergistic interactions between closely spaced target sites, which may account for the potency of some apparently weaker target sites, such as the *Caenorhabditis elegans* lin4-lin14 interaction [32]. In this work, the significant contributions to total Δ*G*_bind-mR_ from the PIWI domain residues (contributing 49.7%) and from the sequence-independent nucleotide 10 (metal ion attraction, contributing 12.0%) strongly suggest that the ‘non-seed’ sites are of high biological relevance (see review in Ref.[11]). Taken together, the data presented here serve to underscore our conclusion that the seed region plays a specifically unique role in the guide-target RNA binding, but is not the only key factor to decide the recognition efficiency. The above findings might answer the question why many bioinformatic target search algorithms overestimate the number of *in vivo* targets, as they are often based on unwarranted assumptions regarding the structure and evolutionary conservation of miRNA seed sites, and no requirements for targeting *in vivo* have been taken into account [11].

### Conclusions

The present MD simulations and thermodynamic analysis provide a new insight into the mechanism of the recognition of Argonaute to miRNA and mRNA at the atomic level. Our findings are summarized as follows:

1. Our MD simulations including PCA clearly addressed the dynamic behavior of the PAZ, Mid and PIWI domains in argonaute protein. Basically, PIWI and Mid domains are rigid and static, whereas PAZ domain is intrinsically flexible. The dynamic mode of PAZ is related not only to the recognition but also to the release of the 3′-end of the guide RNA strand. The movements of the RNA binding domain show more pronounced correlated motions with the substrate binding event, indicating its functional importance.
2. Between the binary and ternary complexes, MD simulations show that difference exists in the geometry and dynamics of the Mg^2+^ interface in PIWI domain that the Mg^2+^ is able to switch between closed and open conformations, which makes the mRNA backbone approach to the catalytic center and form a stable octahedral geometry at the RNA-binding site. Inside the binding pocket of PIWI domain, one water molecule (H1) plays an important role in the conformational changes of Mg^2+^. The physiological importance of this ion atom for target binding is that it contributes above 10% of total ΔG for the binding of mRNA to Ago complex.
3. The thermodynamic analysis shows an arginine-rich containing channel for the RNA binding, accounting for more than 80% of the total ΔG for the miRNA binding to free Ago, and for above 50% for the mRNA to the binary complex. From the energy point of view, the seed region is important, but not as important as the contributions from protein.

Our results provide an improved understanding of above key parameters that define how the guide RNA binds to Ago protein and their target genes. We anticipate that these will be of use in understanding known miRNA–target relationships as well as in improving methods to predict the miRNA targets.

## Materials and Methods

### Molecular models of free Ago and Ago complexes

As RNA always tends to decay in situ, it is difficult to determine the 3D structure of Ago/RNA complex, much less the structure of Ago/miRNA/mRNA complex. To date, Wang et al. have determined the X-ray crystal structures of three argonaute silencing complexes with a seed-containing guide DNA and target RNA duplex (pdb codes: 3F73 [20], 3HM9 [21] and 3DLB [31]), in which DNA-RNA hybrid was used mimicing the miRNA-mRNA complex. In addition, a NMR structure of a let-7:lin-41 complex from *C. elegans* was solved for the miRNA:mRNA complex (pdb code: 2JXV [50]).

In this work, in order to overcome the difficulty of unavailability of miRNA/mRNA/Ago complexes, several modeling and simulation methods were integrated to build the Ago complex structures: 1) A specific miRNA, i.e., miR-7 was selected due to its validated importance for target regulation [28]; The starting B-RNA miR-7 (5′-UGGAAGACUA UGAUUUUGUUGU) and its target mRNA (5′- ACAGCAGAAUCAAUAGUCUUCCG) [28] structures for MD simulations were built using Bioploymer module in Sybyl 6.9 (Tripos Inc. St. Louis, MO) [51], according to the basic features provided by the RNA-target complexes (2JXV.pdb [49] and 3F73.pdb [20]).

Based on the X-ray structure of the Ago silencing complexes (3F73 [20], resolution 3.0 Å, 3HM9 [21], 3.3 Å, 3DLB [31], 2.7 Å), the 3D structural model of Ago was constructed. The side chains with missing coordinates were reconstructed using the fragment library of the Biopolymer module. The bound DNA-RNA fragments were removed from the original structure. Residues around the DNA-binding tunnel within 5 Å, which distance is large enough to include the binding site, were extracted to comprise the binding pocket for docking. Using the protein-protein docking and molecular superposition software Hex 4.2 (http://www.csd.abdn.ac.uk/hex/) [52], and the flexible docking program AutoDock 4 (http://autodock.scripps.edu/) [53], the possibly best binding pose (orientation and conformation) of RNA binding to Ago was searched.

During the docking process, following steps were employed:

1. The crystal structure 3F73.pdb [20] which contains a 5′-phosphorylated 21-nucleotide guide DNA and a 20-nucleotide target RNA was used, with 3DLB.pdb [31] and 3HM9.pdb [21] applied as control. The chain of protein was kept, while other chains for DNA and all water molecules were deleted. During the entire process, however, the active site magnesium ion was maintained (with a charge of +2).
2. By Biopolymer module in Sybyl with default values, the seed duplex of the investigated miR-7 with its target from nucleotides 1–8 (from miRNA 5′ end) was constructed. Then this fragment was docked to the protein binding site with Hex with default parameters, which globally scaned the translational and rotational spaces of the molecules by surface complementarity and an electrostatic filter. From the 500 conformations presented by Hex the final conformation was selected, which successfully reproduced the structure of the complex as judged by superimposing our docked results with the crystal structure of the complex (shown in the Supporting Information Figure S1 (I) and (II) and Dataset S1).
3. The next step is, for each time only two nucleotides were docked, which is an effective procedure developed for efficient DNA/RNA flexible docking [54]. During this process, nucleotides 9–10 (from 3′ end) UA of mRNA, the fully flexible dinucleotides, were firstly docked into the binding site by using Autodock, which is based on a fully flexible ligand-docking algorithm. The pocket the nucleotides UA docked into is defined by the position of the original crystal DNA fragment in 3F73.pdb, and the final active conformation of the ligands was selected according to the same criterion, i.e., the binding modes of the RNA fragment in the above three crystal structures. The most crucial principle in this criterion is that the docking process should assure that, on one hand, the docked phosphate of the fully flexible UA superimposes exactly on the phosphate from the crystal structure's UA, and on the other hand, those key residues correctly binds to the active site magnesium ion in PIWI domain (see Supporting Information Figure S1 (III) and Dataset S1).
  The AutoDock run parameters are summarized as follows: the maximum number of energy evaluations (ga_num_evals) was increased to 2.5×10^7^ per run; the maximum number of generations in the Lamarckian genetic algorithm (ga_num_generations) was increased to 80000; the maximum number of iterations in the pseudo-Solis-and-Wets-minimization/local search (sw_max_its) was increased to 3000. And all other run parameters were maintained at their default settings.
4. For nucleotides 11 to 18 from 3′ end of mRNA, each time a fragment of dinucleotides was docked to the binding channel as revealed by the crystal structures, which process continued until all the fragments were docked. In this way, the four different fully flexible dinucleotides were docked in sequence to the binding domain (Supporting Figure S1 (V and VII)).
5. The rest nucleotides 19–23 of mRNA built by Biopolymer were docked by Hex with default parameters to the PIWI channel. From the millions of docking conformations, the optimal poses were identified by Hex program, resulting in a freely extended 5′ end of mRNA outside of the channel (Supporting Figure S1 (VII)).
6. For docking of all the remaining parts from nucleotides 9 to 23 of the miRNA (miRNA 5′ end) to the binding channel extended from the PIWI to PAZ domain, the same preparatory and AutoDock processes as used for docking of above mRNA to the protein were applied. For each step, the most important thing is to make sure that the docked phosphate of the dinucleotides is embedded in the receptor binding channel as revealed by the RNA binding modes in the crystal structures (3F73, 3DLB and 3HM9) (Supporting Figure S1 (IV) & (V)). The detailed information of the binding modes of ligands to Ago can be seen in supporting Dataset S1, which shows the superposition of docked model with 3F73.pdb.

The binary structure was obtained by removing the mRNA from the ternary system. Subsequently, the structures were treated with a rigid-body energy minimization in Hex to further optimize the pose, followed by molecular dynamics simulations.

### MD simulations

All molecular dynamics simulations were carried out using the GROMACS 4.0 package [55] on a 2.2 GHz 28 Intel Xeon workstation with 4×11 Gb of RAM with the AMBER99 force-field [56]. For MD simulations, three models (free Ago, Ago-miRNA, and Ago-miRNA-mRNA complexes) were solvated with the TIP3P water model [57]. The V-rescale thermostat [58] was applied using a coupling time of 0.1 ps to maintain the systems at a constant temperature of 300K, with pressure maintained by coupling to a reference pressure of 1 bar, and values of the isothermal compressibility set to 4.5×10^−5^ bar^−1^ for water simulations. Periodic boundary conditions were employed and Particle Mesh Ewald [59] was used for the long-range electrostatic interactions. Van der Waals and Coulomb interactions were truncated at 1.4 and 1.0 nm, respectively. All bond lengths including hydrogen atoms were constrained by the LINCS algorithm [60].

For each system, the simulation cell was a cubic periodic box with a side length of 106 Å, and the minimum distance between the protein and the box wall was set to be larger than 10 Å so that the protein does not directly interact with its own periodic image given the cutoff in every system. Numerical integration of the equations of motion used a time step of 2 fs, with non-bonded pair list updated every 10 steps and conformations stored every 2 ps for analysis.

To neutralize the total charge and bring the tonicity of the solvent to physiological levels of 0.15 M, 107 Na^+^ and 109 Cl^−^, 107 Na^+^ and 91 Cl^−^, and 108 Na^+^ and 70 Cl^−^ ions were placed randomly in the boxes of free Ago, Ago-miRNA and Ago-miRNA-mRNA complexes, respectively. In the end, the simulation system for free Ago is totally composed of 117492 atoms, with 10737 protein atoms and 35513 solvent molecules embedded in the cubic box. While the binary system (Ago-miRNA) contains a total of 117468 atoms, including 10737 protein atoms, 735 RNA atoms and 35266 solvent atoms. And the ternary system (Ago-miRNA-mRNA) is consisted of 12209 solution atoms and 35409 solvent atoms.

After initial configuration construction, a standard equilibration protocol was performed for molecular simulations. The system was subjected to energy minimization for 5000 steps by steepest descent and for 15000 steps by conjugate gradient to avoid close atomic contacts, followed by slow constant volume heating to 300 K over 100 ps using 2.4 kcal/mol/Å^2^ harmonic restraints. These restraints were slowly reduced to zero during a series of energy minimizations and 50 ps equilibration steps at constant temperature (300 K) and pressure (1 bar) with a 0.2 ps coupling constant for both parameters. The final equilibration step was a 100 ps constant volume run. The production stage consisted of a total of 20 ns at constant temperature of 300 K for all the three systems, respectively.

### Principal component analysis

In order to identify the most significant fluctuation modes of the protein and RNAs, principal component analysis was carried out essentially as described by Wlodek et al [61]. The positional covariance matrix *C* was calculated from the equilibrated portion of each trajectory. For *M* snapshots of an *N* atom system, *C* is a 3*N*×3*N* matrix:
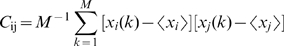
(1)The eigenvectors of the covariance matrix, V, together with their corresponding eigenvalues, *λ*, were obtained by diagonalizing the covariance matrix *C*, i.e., 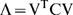. The (orthonormal) eigenvectors provide a 3*N*-dimensional representation of principal modes of structural variation, and the eigenvalue for a mode indicates the relative contribution that this mode has made to motion within the trajectory, as calculated by 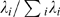. In the rotated Cartesian coordinate, the largest eigenvalue *λ* captures the largest fraction of the relative mean square fluctuation (RMSF), and the second largest *λ* captures the next largest fraction of the RMSF, etc. Projections of the trajectory (*r(t)*) on major eigenvectors (Eq.(2)) were analyzed for their time-dependent behavior and probability distributions [62].
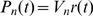
(2)The domain motion analyses based on the PCA results were performed using the DYNDOM program [29].

### Binding free energy calculation

The binding free energy [63] was calculated by Eq. (3) as follows:
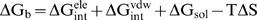
(3)where Δ*G*_b_ is the binding free energy in solution, Δ*G*_int_^ele^ and ΔG_int_^vdw^ are electrostatic and van der Waals interaction energies between a protein and its ligand, respectively, Δ*G*_sol_ is the solvation energy, and −*T*Δ*S* is the contribution of conformational entropy to the binding. Δ*G*_int_^ele^ and Δ*G*_int_^vdw^ were computed using the same parameter set as used in the MD simulation, with no cutoff applied to the calculation.

Solvation energy Δ*G*_sol_ was calculated using the MM/PBSA scripts supplied with AMBER 10.0 [64]. The free energy estimation involves separately evaluating the free energy for the solute and solvent for a series of snapshots and then averaging the results. Ensembles of the structures (500 snapshots) for the MM/PBSA calculation were obtained from 6 ns MD simulations of the solvated complex.

The electrostatic component of the solvation was estimated with a Poisson-Boltzmann electrostatic continuum method using program Delphi II [65]. The dielectric boundary is the molecular surface defined by a 1.4 Å probe sphere and by spheres centered on each atom with radii taken from the PARSE parameter set [66] 1.4 Å, with a value of 2.0 Å for the phosphorus. An interior dielectric of unity was used (matching the dielectric chosen when evaluating the solute electrostatic interactions to scale the charges [67], [68]) and the outside dielectric was set to 80. The nonpolar solvation energy was calculated from the solvent-accessible surface area (SASA), obtained with the Amber molsurf module using a probe radius of 1.4 Å [69] with a parametrization of
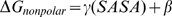
(4)where *γ* = 0.00542 kcal/Å^2^ mol and *β* = 0.92 kcal/mol.

Estimated by a normal-mode analysis of the vibration frequencies, the entropy was calculated with Amber nmode module. This normal mode calculation is extremely time-consuming for large systems, thus only residues within 9 Å of the mass center of the ligand (including the ligand, but excluding water molecules) were used for the normal mode calculation [70]. Using a distance-dependent dielectric constant of *ε* = 4*r*, the truncated systems were minimized until the root-mean-square of the elements of the gradient vector was less than 5×10^−5^ kcal mol^−1^ Å^−1^, and then the entropies were calculated using classical statistical formula [71]. 100 snapshots (every fifth snapshot of 500 collected snapshots) were used to estimate the contribution of the entropies of association [70].

The ligand-residue interaction decomposition was also performed by decomposing the total binding free energy into the contribution from each individual residue by the MM/GBSA method [72], since PB energy decomposition is rather time-consuming and computationally expensive. The binding interaction of each ligand-residue pair includes three terms (the entropy term is omitted because of its relative small contributions): electrostatic contribution (Δ*G*_ele_), van der Waals contribution (Δ*G*_vdw_) and solvation contribution (Δ*G*_sol_). All energy components were calculated using 100 snapshots from 500 collected snapshots.

## Supporting Information

Dataset S1 All_alignment.pdb, the superposition of the initial docked structure with 3F73.pdb (This pdb structure can be viewed by pymol sofware).
(0.80 MB ZIP)

Dataset S2 Ago.pdb, the average structure of the simulated ternary system of miRNA-mRNA-Ago.
(0.17 MB ZIP)

Figure S1 Superposition of docked structure with the crystal 3F73.pdb. (I). Superposition of seed fragment of the X-ray structure with the docked model. The cyan and gray represent the crystal structures of 3F73.pdb and Ago model used for docking respectively. Orange ribbons represent the DNA duplex in the protein. Yellow (miRNA) and red (mRNA) ribbons represent the docked seed fragment. This figure shows that docked structure is well overlapped with the crystal structure. (II). Mg 681 (pink sphere) well interacts with the docked RNA as in the crystal structure. (III). Mg 679 (green sphere) well interacts with U717 as in the crystal structure. (IV). Superposition of the miRNA (yellow) 3′ end with the crystal DNA fragment (orange). Some nucleotides of the crystal DNA fragment are missing as shown by the non-continuous orange ribbons. (V). The overall view of the superposition of the docked structure after minimization by Hex with the crystal structure 3F73.pdb. (VI). Extension of the docked model for 9–10 nucleotides for mRNA. (VII). Extension of the 11–23 nucleotides for mRNA.
(0.89 MB TIF)

Figure S2 RMS deviations with respect to the starting structure in the simulation of the three species of free Ago, Ago-miRNA and Ago-miRNA-mRNA.
(0.07 MB JPG)

Figure S3 The root-mean square fluctuation (RMSF) of Cα atoms of Ago in the three structures. PAZ (amino acids: 20–100, 170–260), PIWI (amino acids: 463–678), and Mid (amino acids: 326–462).
(0.04 MB JPG)

Figure S4 The structure model of Ago ternary complex.
(0.44 MB TIF)

Figure S5 All-atom root-mean-squared deviation to the starting structure of seed segment (2–8) for the unbounded miRNA and bounded miRNA-mRNA.
(0.04 MB JPG)

Figure S6 RMSD of the Mg^2+^-binding interface in (A) mRNA-free and (B) mRNA-bound simulations, respectively. Red: single Mg679; Green: Mg679 interface atoms (7 molecules: Mg^2+^, four O atoms, and two water molecules at H1 and H2 sites); Black: single Mg680; Blue: Mg680 interface atoms (4 molecules).
(0.07 MB JPG)

Table S1 Principal component analysis of the simulation trajectories of the ternary, binary and single systems for Ago Cα.
(0.05 MB DOC)

Table S2 Principal component analysis of the simulation trajectories of the ternary and binary systems for miRNA.
(0.04 MB DOC)

Table S3 Principal component analysis of the simulation trajectories of the ternary systems for mRNA.
(0.04 MB DOC)

Table S4 Hinge parameters of the three systems by PCA.
(0.05 MB DOC)

Table S5 Main hydration sites observed during simulations of the binary (miRNA-Ago) and ternary (miRNA-mRNA-Ago) systems.
(0.03 MB DOC)
