# Supplementary figures and images for: Mechanism of MicroRNA-Target Interaction: Molecular Dynamics Simulations and Thermodynamics Analysis
Source: PLoS Comput Biol. 2010 Jul 29;6(7):e1000866. doi: 10.1371/journal.pcbi.1000866 (PMC2912339; doi:10.1371/journal.pcbi.1000866)

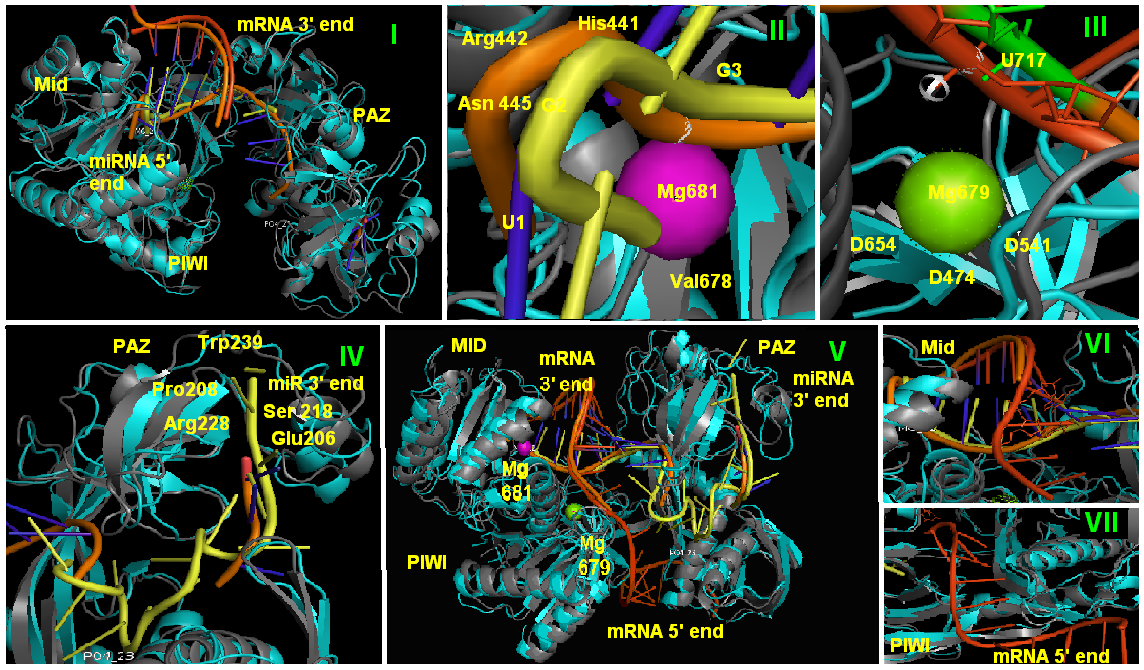

Supplement: Figure S1 — Superposition of docked structure with the crystal 3F73.pdb. (I). Superposition of seed fragment of the X-ray structure with the docked model. The cyan and gray represent the crystal structures of 3F73.pdb and Ago model used for docking respectively. Orange ribbons represent the DNA duplex in the protein. Yellow (miRNA) and red (mRNA) ribbons represent the docked seed fragment. This figure shows that docked structure is well overlapped with the crystal structure. (II). Mg 681 (pink sphere) well interacts with the docked RNA as in the crystal structure. (III). Mg 679 (green sphere) well interacts with U717 as in the crystal structure. (IV). Superposition of the miRNA (yellow) 3′ end with the crystal DNA fragment (orange). Some nucleotides of the crystal DNA fragment are missing as shown by the non-continuous orange ribbons. (V). The overall view of the superposition of the docked structure after minimization by Hex with the crystal structure 3F73.pdb. (VI). Extension of the docked model for 9–10 nucleotides for mRNA. (VII). Extension of the 11–23 nucleotides for mRNA. (0.89 MB TIF) [file pcbi.1000866.s003.tif]

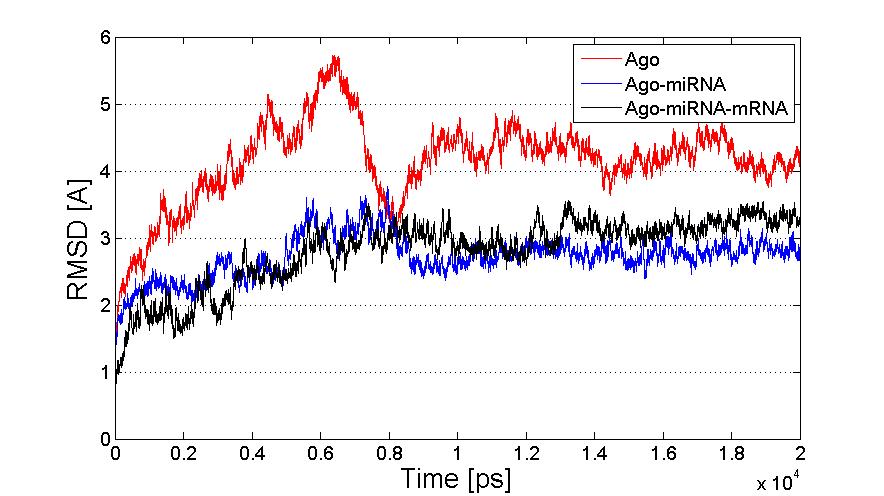

Supplement: Figure S2 — RMS deviations with respect to the starting structure in the simulation of the three species of free Ago, Ago-miRNA and Ago-miRNA-mRNA. (0.07 MB JPG) [file pcbi.1000866.s004.jpg]

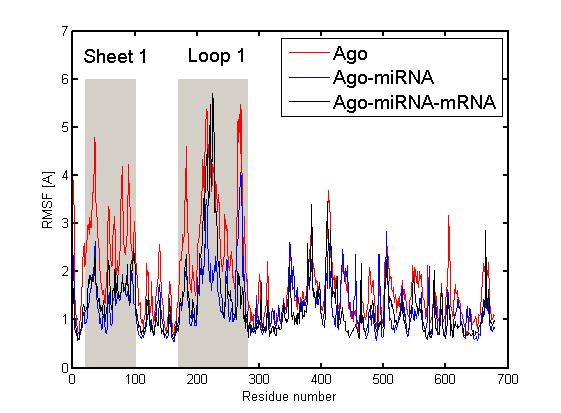

Supplement: Figure S3 — The root-mean square fluctuation (RMSF) of Cα atoms of Ago in the three structures. PAZ (amino acids: 20–100, 170–260), PIWI (amino acids: 463–678), and Mid (amino acids: 326–462). (0.04 MB JPG) [file pcbi.1000866.s005.jpg]

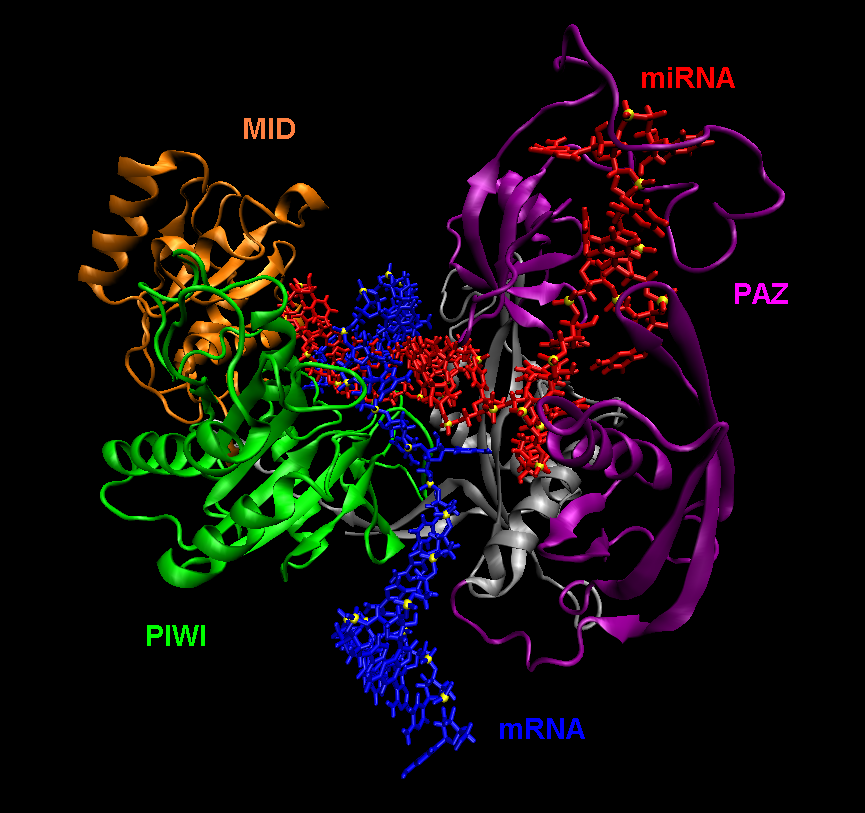

Supplement: Figure S4 — The structure model of Ago ternary complex. (0.44 MB TIF) [file pcbi.1000866.s006.tif]

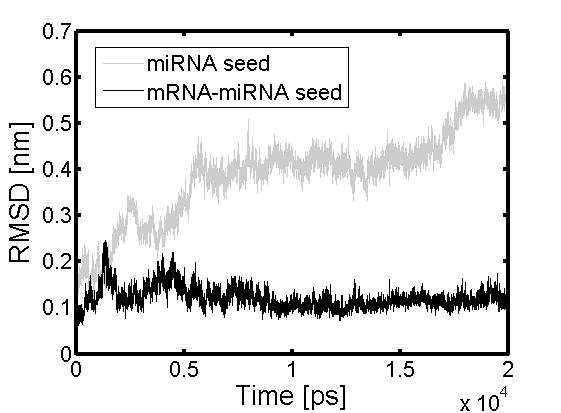

Supplement: Figure S5 — All-atom root-mean-squared deviation to the starting structure of seed segment (2–8) for the unbounded miRNA and bounded miRNA-mRNA. (0.04 MB JPG) [file pcbi.1000866.s007.jpg]

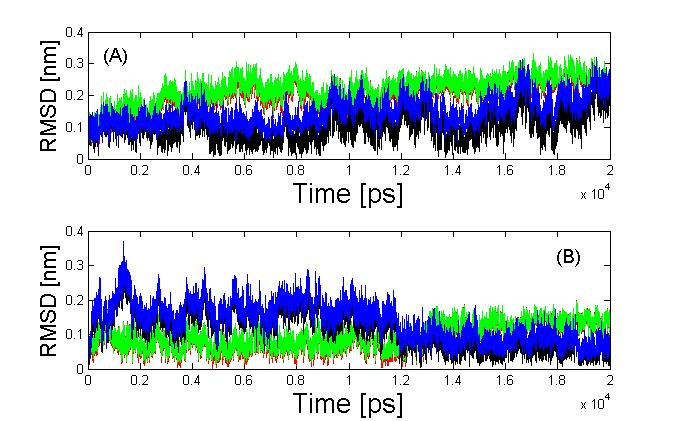

Supplement: Figure S6 — RMSD of the Mg2+-binding interface in (A) mRNA-free and (B) mRNA-bound simulations, respectively. Red: single Mg679; Green: Mg679 interface atoms (7 molecules: Mg2+, four O atoms, and two water molecules at H1 and H2 sites); Black: single Mg680; Blue: Mg680 interface atoms (4 molecules). (0.07 MB JPG) [file pcbi.1000866.s008.jpg]
