# Supplementary material for: Mechanism of MicroRNA-Target Interaction: Molecular Dynamics Simulations and Thermodynamics Analysis
Source: PLoS Comput Biol. 2010 Jul 29;6(7):e1000866. doi: 10.1371/journal.pcbi.1000866 (PMC2912339; doi:10.1371/journal.pcbi.1000866)
Supplement: Table S1 — Principal component analysis of the simulation trajectories of the ternary, binary and single systems for Ago Cα. (0.05 MB DOC) [file pcbi.1000866.s009.doc]

***Table S1.*** Principal component analysis of the simulation trajectories of the ternary, binary and single systems for Ago Cα

|  | Ternary | | Binary | | Single | | |
| --- | --- | --- | --- | --- | --- | --- | --- |
| Number of atom | 678 | | 678 | | 678 | | |
| Number of eigenvectors | 10 | | 10 | | 10 | | |
| Total variance | 12.1935 | | 8.3848 | | 31.0822 | | |
| Explained variance | 9.2869 | | 5.5833 | | 26.7480 | | |
| Quality of the compression | 76.2% | | 66.6% | | 86.1% | | |
| Eigenvectors | Eigenvalues/Weight (%) | | | | | | |
| 1 | 5.3559 | 43.92 | 1.7492 | 20.86 | | 12.6665 | 40.75 |
| 2 | 1.2763 | 10.47 | 1.1504 | 13.72 | | 5.3395 | 17.18 |
| 3 | 0.8376 | 6.87 | 0.7136 | 8.51 | | 3.3807 | 10.88 |
| 4 | 0.4166 | 3.42 | 0.6078 | 7.25 | | 1.9112 | 6.15 |
| 5 | 0.3930 | 3.22 | 0.4400 | 5.25 | | 1.0964 | 3.53 |
| 6 | 0.2742 | 2.25 | 0.2445 | 2.92 | | 0.7227 | 2.33 |
| 7 | 0.2442 | 2.00 | 0.2032 | 2.42 | | 0.5890 | 1.90 |
| 8 | 0.2080 | 1.71 | 0.1724 | 2.06 | | 0.4720 | 1.52 |
| 9 | 0.1523 | 1.25 | 0.1590 | 1.90 | | 0.3026 | 0.97 |
| 10 | 0.1288 | 1.06 | 0.1432 | 1.71 | | 0.2674 | 0.86 |
