# Supplementary material for: Mechanism of MicroRNA-Target Interaction: Molecular Dynamics Simulations and Thermodynamics Analysis
Source: PLoS Comput Biol. 2010 Jul 29;6(7):e1000866. doi: 10.1371/journal.pcbi.1000866 (PMC2912339; doi:10.1371/journal.pcbi.1000866)
Supplement: Table S2 — Principal component analysis of the simulation trajectories of the ternary and binary systems for miRNA. (0.04 MB DOC) [file pcbi.1000866.s010.doc]

***Table S2.*** Principal component analysis of the simulation trajectories of the ternary and binary systems for miRNA

|  | Ternary | | Binary | |
| --- | --- | --- | --- | --- |
| Number of atom | 733 | | 733 | |
| Number of eigenvectors | 10 | | 10 | |
| Total variance | 14.4373 | | 16.4477 | |
| Explained variance | 11.9655 | | 12.4750 | |
| Quality of the compression | 82.9% | | 75.8% | |
| Eigenvectors | Eigenvalues/Weight (%) | | | |
| 1 | 7.7431 | 53.63 | 5.2559 | 31.96 |
| 2 | 1.6575 | 11.48 | 2.5439 | 15.47 |
| 3 | 0.7189 | 4.98 | 1.5386 | 9.35 |
| 4 | 0.4955 | 3.43 | 0.9322 | 5.67 |
| 5 | 0.3624 | 2.51 | 0.4909 | 2.98 |
| 6 | 0.3084 | 2.14 | 0.4718 | 2.87 |
| 7 | 0.2047 | 1.42 | 0.3738 | 2.27 |
| 8 | 0.1759 | 1.22 | 0.3225 | 1.96 |
| 9 | 0.1577 | 1.09 | 0.2771 | 1.68 |
| 10 | 0.1415 | 0.98 | 0.2683 | 1.63 |
