# Supplementary material for: Mechanism of MicroRNA-Target Interaction: Molecular Dynamics Simulations and Thermodynamics Analysis
Source: PLoS Comput Biol. 2010 Jul 29;6(7):e1000866. doi: 10.1371/journal.pcbi.1000866 (PMC2912339; doi:10.1371/journal.pcbi.1000866)
Supplement: Table S3 — Principal component analysis of the simulation trajectories of the ternary systems for mRNA. (0.04 MB DOC) [file pcbi.1000866.s011.doc]

***Table S3.*** Principal component analysis of the simulation trajectories of the ternary systems for mRNA

|  | mRNA | |
| --- | --- | --- |
| Number of atom | 735 | |
| Number of eigenvectors | 10 | |
| Total variance | 85.3650 | |
| Explained variance | 79.6298 | |
| Quality of the compression | 93.3% | |
| Eigenvectors | Eigenvalues/Weight (%) | |
| 1 | 37.1782 | 43.55 |
| 2 | 22.1489 | 25.95 |
| 3 | 10.0363 | 11.76 |
| 4 | 3.0761 | 3.60 |
| 5 | 2.3069 | 2.70 |
| 6 | 1.7633 | 2.07 |
| 7 | 1.2061 | 1.41 |
| 8 | 0.8548 | 1.00 |
| 9 | 0.6012 | 0.70 |
| 10 | 0.4581 | 0.54 |
