# Supplementary material for: Mechanism of MicroRNA-Target Interaction: Molecular Dynamics Simulations and Thermodynamics Analysis
Source: PLoS Comput Biol. 2010 Jul 29;6(7):e1000866. doi: 10.1371/journal.pcbi.1000866 (PMC2912339; doi:10.1371/journal.pcbi.1000866)
Supplement: Table S4 — Hinge parameters of the three systems by PCA. (0.05 MB DOC) [file pcbi.1000866.s012.doc]

***Table S4.*** Hinge parameters of the three systems by PCA

| Ago system | Fixed domain | Moving domain | Rotation angle | Translation (A) | Closure  (%) | Bending residues |
| --- | --- | --- | --- | --- | --- | --- |
| Single | 1 | Hinge 1 | 25.6 | -0.4 | 84.4 | 178-179 189-190 199-200  253-254 |
|  | 1 | Hinge 2 | 14.5 | -0.5 | 52.4 | 15-16 53-54 57-58 89-90  154-155 157-158 |
|  | 1 | Hinge 3 | 20.5 | -0.9 | 68.5 | 126-127 148-149 162-163  272-279 |
|  | 1 | Hinge 4 | 19.3 | -0.2 | 6.8 | 4 - 5 8 - 10  301 - 302 306 - 310 316 - 317 572 - 576 583 - 584 588 - 591 608 - 610 |
| Binary | 1 | Hinge 5 | 11.9 | 0.4 | 19.6 | 309-310 |
|  | 1 | Hinge 6 | 11.3 | -0.5 | 94.2 | 172 - 173 177 - 181 183 - 189 196 - 197 199 - 205 239 - 241 247 - 251 257 - 258 260 - 261 267 - 268 |
|  | 1 | Hinge 7 | 12.0 | 0.6 | 35.2 | 311 - 312 457 - 458 498 - 508 634 - 637 642 - 643 661 - 662 671 - 672 675 - 676 |
| Ternary | 1 | Hinge 8 | 24.9 | 1.5 | 82.6 | 206 - 211 235 - 236 |
|  | 1 | Hinge 9 | 12.5 | 0.7 | 82.4 | 304 - 305 |
|  | 1 | Hinge 10 | 12.9 | 0.1 | 99.7 | 12 - 13 16 - 17  20 - 21 53 - 55  60 - 61 63 - 67  69 - 70 90 - 102 104 - 105 120 - 121 152 - 153 159 - 160 292 - 293 299 - 306 |
|  | 1 | Hinge 11 | 8.9 | 0.1 | 84.3 | 130 - 131 144 - 145 164 - 165 167 - 168 170 - 171 258 - 259 261 - 262 |
