# Supplementary material for: Mechanism of MicroRNA-Target Interaction: Molecular Dynamics Simulations and Thermodynamics Analysis
Source: PLoS Comput Biol. 2010 Jul 29;6(7):e1000866. doi: 10.1371/journal.pcbi.1000866 (PMC2912339; doi:10.1371/journal.pcbi.1000866)
Supplement: Table S5 — Main hydration sites observed during simulations of the binary (miRNA-Ago) and ternary (miRNA-mRNA-Ago) systems. (0.03 MB DOC) [file pcbi.1000866.s013.doc]

***Table S5.*** Main hydration sites observed during simulations of the binary (miRNA-Ago) and ternary (miRNA-mRNA-Ago) systems

| Site | Substrates | Occupancy | Residence time (ns) |
| --- | --- | --- | --- |
| H1 | Mg, F473, L649 | 100% | 0.1-20 |
| H2 (binary) | Mg | 100% | 0.1-20 |
| H2 (ternary) | Mg, U717-P | 100% | 0.1-20 |
| H3 | D474 | 50% | 5-20 |
| H4 | D653 | 60% | 4-20 |
| H5 | D541 | 100% | 0.2-20 |
| H6 | D541 | 100% | 0.1-20 |
